# Differential Effects of Iron, Zinc, and Copper on *Dictyostelium discoideum* Cell Growth and Resistance to *Legionella pneumophila*

**DOI:** 10.3389/fcimb.2017.00536

**Published:** 2018-01-11

**Authors:** Simona Buracco, Barbara Peracino, Claudia Andreini, Enrico Bracco, Salvatore Bozzaro

**Affiliations:** ^1^Department of Clinical and Biological Sciences, University of Torino, Turin, Italy; ^2^Magnetic Resonance Center (CERM), University of Florence, Florence, Italy; ^3^Department of Oncology, University of Torino, Turin, Italy

**Keywords:** *Dictyostelium discoideum*, *Legionella pneumophila*, host-pathogen interactions, phagocytosis, Nramp1, NrampB, divalent metals, Zinpyr-1

## Abstract

Iron, zinc, and copper play fundamental roles in eucaryotes and procaryotes, and their bioavailability regulates host-pathogen interactions. For intracellular pathogens, the source of metals is the cytoplasm of the host, which in turn manipulates intracellular metal traffic following pathogen recognition. It is established that iron is withheld from the pathogen-containing vacuole, whereas for copper and zinc the evidence is unclear. Most infection studies in mammals have concentrated on effects of metal deficiency/overloading at organismal level. Thus, zinc deficiency or supplementation correlate with high risk of respiratory tract infection or recovery from severe infection, respectively. Iron, zinc, and copper deficiency or overload affects lymphocyte proliferation/maturation, and thus the adaptive immune response. Whether they regulate innate immunity at macrophage level is open, except for iron. The early identification in a mouse mutant susceptible to mycobacterial infection of the iron transporter Nramp1 allowed dissecting Nramp1 role in phagocytes, from the social amoeba *Dictyostelium* to macrophages. Nramp1 regulates iron efflux from the phagosomes, thus starving pathogenic bacteria for iron. Similar studies for zinc or copper are scant, due to the large number of copper and zinc transporters. In *Dictyostelium*, zinc and copper transporters include 11 and 6 members, respectively. To assess the role of zinc or copper in *Dictyostelium*, cells were grown under conditions of metal depletion or excess and tested for resistance to *Legionella pneumophila* infection. Iron shortage or overload inhibited *Dictyostelium* cell growth within few generations. Surprisingly, zinc or copper depletion failed to affect growth. Zinc or copper overloading inhibited cell growth at, respectively, 50- or 500-fold the physiological concentration, suggesting very efficient control of their homeostasis, as confirmed by Inductively Coupled Plasma Mass Spectrometry quantification of cellular metals. *Legionella* infection was inhibited or enhanced in cells grown under iron shortage or overload, respectively, confirming a major role for iron in controlling resistance to pathogens. In contrast, zinc and copper depletion or excess during growth did not affect *Legionella* infection. Using Zinpyr-1 as fluorescent sensor, we show that zinc accumulates in endo-lysosomal vesicles, including phagosomes, and the contractile vacuole. Furthermore, we provide evidence for permeabilization of the *Legionella*-containing vacuole during bacterial proliferation.

## Introduction

Transition metals, such as iron, zinc, copper, or manganese, play fundamental roles in many biological processes in both procaryotes and eucaryotes. Thanks to their ability to easily shift between different oxidation states, they act as co-factors of enzymes, and it is estimated that 30–45% of known enzymes are metalloproteins containing one of these metals (Andreini et al., 2008; Waldron et al., 2009). Among the transition metals, iron is the most abundant in living organisms. By shifting between the ferric and the ferrous forms, iron catalyzes redox reactions that are essential for processes such as respiration, oxygen transport, metabolic energy production, and gene regulation (Andreini et al., 2008; Pantopoulos et al., 2012). Whereas, iron is mostly present in the environment as insoluble ferric form, copper is easily bioavailable in the soluble Cu^2+^ cupric form (Festa and Thiele, 2011). Similarly to iron, copper is involved in redox reactions, regulating the activity of enzymes, such as the respiratory chain cytochrome c oxidase and the Cu-Zn superoxide dismutase (Tapiero and Tew, 2003; Rubino and Franz, 2012). Zinc is the second most abundant transition metal in living organisms after iron, is incorporated in about 10% of human proteins and necessary for over 300 enzymes (Andreini et al., 2011). In contrast to iron and copper, zinc is redox-inert, but has many structural and catalytic roles, stabilizing negative charges of the substrates or organizing protein subdomains in zinc motifs (Tapiero and Tew, 2003; Cerasi et al., 2013; Maret, 2013).

Excess iron, copper, or zinc are toxic, as they perturb the redox potential, producing highly reactive hydroxyl radicals (copper and iron), bind to sulfide and thiol groups, thus destabilizing iron-sulfur clusters (copper and zinc), or interfere with the metabolism of other ions, displacing them from their binding proteins (Letelier et al., 2005; Dupont et al., 2011; Imlay, 2014). Thus, cells have developed complex mechanisms to regulate import, export, and storage of transition metals (Stafford et al., 2013; Bird, 2015; Weiss and Carver, 2017).

Tight regulation of transition metal bioavailability is also a vital part of host-pathogen interactions. In bacteria, transition metals are involved in metabolism and regulation of virulence as a mechanism of host invasion, and many opportunistic pathogens, such as pathogenic strains of *Escherichia* or *Klebsiella, Mycobacteria, Salmonella*, or *Legionella*, have developed sophisticated sensing, uptake, and export mechanisms to accumulate transition metals according to their physiological needs (Graham et al., 2009; Dupont et al., 2011; Rowland and Niederweis, 2012; Porcheron et al., 2013; Braymer and Giedroc, 2014; Neyrolles et al., 2015; Skaar and Raffatellu, 2015; Capdevila et al., 2016).

The source of transition metals for intracellular pathogens is the cytoplasm of the host cell, which in turn can manipulate metal uptake and intracellular traffic following pathogen recognition. Several lines of evidence support the notion that iron and manganese are withheld from the phagosome, or the pathogen-containing vacuole, to prevent reconstruction by the engulfed bacteria of Fe-S clusters and the use of Mn^2+^ as a protectant against reactive oxygen species (Weinberg, 2000; Kehres and Maguire, 2003; Nairz et al., 2010; Lisher and Giedroc, 2013). Iron efflux from phagosomes by the Nramp1 transporter, both in mammalian macrophage and the lower professional phagocyte *Dictyostelium discoideum*, is such a paradigmatic case of starvation strategy, or so-called “nutritional immunity” (Appelberg, 2006; Cellier, 2012; Bozzaro et al., 2013a). In contrast to iron, copper, and zinc are presumably used as a mechanism to poison bacterial pathogens (Wagner et al., 2005; White et al., 2009; Botella et al., 2012; Soldati and Neyrolles, 2012; Djoko et al., 2015), though zinc sequestration as antibacterial weapon has also been described (Stafford et al., 2013; Vignesh et al., 2013; Djoko et al., 2015; Besold et al., 2016). Copper withdrawal has been shown to play a role for resistance to fungal pathogens, raising the possibility that it may be used also for bacteria (Besold et al., 2016). Except for iron, most studies with mammals provide only indirect evidence of the role of copper or zinc in immune defense, while an understanding of the underlying processes at macrophage level is lacking or very limited (White et al., 2009). Indeed, most infection studies have concentrated on microbial mechanisms of defense against metal poisoning or on the effects of metal deficiency or overload at organismal level, both in laboratory animals and in epidemiological studies with humans (Prohaska and Lukasewycz, 1981; Chaturvedi and Henderson, 2014; Prasad, 2014; Pasricha and Drakesmith, 2016). These studies have shown the importance of zinc or copper for several physiological processes, including proper development of the immune system, lymphocyte differentiation, and adaptive immunity (Bonaventura et al., 2015; Weiss and Carver, 2017). Their role in cell-autonomous defense mechanisms, i.e., innate immunity, at the level of monocytes or macrophages, is less clear. It has been shown that both free zinc and copper accumulate in endosomal vesicles, including phagosomes, in response to inflammatory signals and mycobacterial infection (Wagner et al., 2005; Botella et al., 2011), and that silencing of the Cu-ATPase ATP7A impairs macrophage bactericidal activity (White et al., 2009). Enhanced expression of a mycobacterial P-type ATPase, CtpC, which favors zinc efflux, has been also reported, with a *ctpc*-null mutant growing poorly in macrophages (Botella et al., 2011).

For assessing the role of zinc or copper, macrophage cell lines or explanted monocytes could be grown in defined serum-free media, which contain, however, traces of transition metals in addition to proprietary components. Addition of extracellular or intracellular metal chelators to circumvent this limitation often fails, as they are not specific for a given metal and may have other unknown effects, stripping metals from exposed cellular proteins (Kay, 2004). A valid alternative is the use of single-celled amoebae or protozoa, which are the natural hosts of many bacterial pathogens that occasionally infect animals or humans. It is generally recognized that the ability of many bacterial pathogens to grow in macrophages and cause animal or human diseases is a consequence of their adaptation and survival in the normally hostile amoeboid niche, which is the training ground for evolution of virulence traits (Greub and Raoult, 2004; Casadevall, 2008; Cosson and Soldati, 2008; Salah et al., 2009; Erken et al., 2013; Tosetti et al., 2014). An established amoeboid model organism for phagocytosis and host-pathogen interactions is the social amoeba *D. discoideum*. *Dictyostelium* cells are free-living soil amoebae that grow by engulfing and digesting bacteria, and as such they are potential hosts of pathogens (Bozzaro et al., 2008, 2013a; Cosson and Soldati, 2008). Being haploid and amenable to molecular genetic techniques, *Dictyostelium* offers many advantages for identifying and characterizing host genes involved in resistance to pathogens (Bozzaro and Eichinger, 2011). Studies in the last decade have shown that *Dictyostelium* cells share with mammalian macrophages not only the basic phagocytic machinery, but also many mechanisms of innate and nutritional immunity (Bozzaro et al., 2008, 2013a; Cosson and Soldati, 2008; Soldati and Neyrolles, 2012; Nasser et al., 2013; Gaudet et al., 2016). Concerning transition metals, *Dictyostelium* cells share with macrophages the expression of the Nramp1 iron transporter in the phagolysosome, which is essential for proton-driven iron efflux from the phagosome, thus potentially starving bacteria for iron and manganese (Forbes and Gros, 2003; Courville et al., 2006; Peracino et al., 2006; Buracco et al., 2015). In agreement with this function, *nramp1* KO mutants display increased susceptibility to infection by *L. pneumophila* and *M. avium* (Peracino et al., 2006). *L. pneumophila* was also shown to hinder H^+^ V-ATPase, but not Nramp1, recruitment to the *Legionella*-containing macropinosome, thus manipulating Nramp1 activity (Peracino et al., 2010). *Dictyostelium* is also unique among amoebae and protozoa, for encoding in the genome a second Nramp protein, NrampB (formerly Nramp2), belonging to the prototypical Nramp family (Courville et al., 2006; Peracino et al., 2013). NrampB, is expressed in the membrane of the contractile vacuole, and, together with Nramp1, appears to regulate iron homeostasis by transporting iron across the membrane of the contractile vacuole. Mutants defective in NrampB display also increased susceptibility to *Legionella*, suggesting that perturbation of iron homeostasis affects host-pathogen interactions (Bozzaro et al., 2013a).

The *Dictyostelium* genome encodes three SLC31 (CTR) copper transporters, and three P-type Cu-ATPases, one of which is a homolog of the human ATP7A P-type ATPase (The Dictyostelium webpage: http://www.dictybase.org). Both ATP7A and the CTR protein p80 are localized in the plasma membrane and transitorily in phagosomes (Ravanel et al., 2001; Burlando et al., 2002; Hagedorn and Soldati, 2007). ATP7A activity in the plasma membrane is apparently responsible for the refractoriness of *Dictyostelium* cells to high copper concentrations in medium (Burlando et al., 2002; Balbo and Bozzaro, 2008), whereas its transient recruitment to the phagosomal membrane points to a potential involvement in pumping copper in the phagosomal lumen, favoring a potential toxic effect of this metal on bacteria (Hao et al., 2016). The p80 copper transporter could, instead, be involved in copper efflux from the phagosome, but no functional studies have been done in this regard. The zinc transporter family includes 11 members, with seven ZIP and four ZNT family members (Sunaga et al., 2008; The Dictyostelium webpage: http://www.dictybase.org), but no data are available on their localization in phagosome and their potential involvement in host-pathogen interactions.

To assess a role for zinc or copper in *Dictyostelium* phagocytosis and defense mechanisms against bacterial pathogens, and given the large number of transporters for these metals, we have followed in this paper a holistic approach, based on cultivation of wild type cells or Nramp1 knockout mutant in a minimal medium depleted of, or overloaded with either zinc, copper, or iron. The rationale is that extensive growth in media deprived of or with high content of a given metal, should results in either metal deficiency or overload in cells, thus potentially altering their resistance to pathogens. We show here that iron, but not copper or zinc deficiency affects cell growth. Metal excess results in inhibition of cell growth, with the cells being much more sensitive to iron than to copper or zinc overloading. Cells grown under iron depleting or overloading conditions, show, respectively, resistance to or exacerbation of *L. pneumophila* infection, whereas *Legionella* intracellular growth is unaltered by depletion or overloading with copper or zinc. Thus, it appears that zinc and copper, in contrast to iron, play a minor role in *Dictyostelium* resistance against *Legionella*. We provide also evidence for very tight regulation of copper and zinc homeostasis in *Dictyostelium*, which can in part explain these results, and for accumulation of free zinc ions in vesicles of the endo-lysosomal pathway and in the contractile vacuole.

## Materials and methods

### Cell and bacterial strains and culture methods

*Dictyostelium discoideum* parental strain AX2 and the Nramp1-KO mutant were used. The Nramp1-KO mutant or cells producing Nramp1-RFP and NrampB-RFP were generated previously in the lab (Peracino et al., 2006; Buracco et al., 2015). AX2 cells producing calnexin-GFP (Müller-Taubenberger, 2001) and the 389-2 vector for the expression of mRFPmars (Fischer et al., 2004) were provided by Annette Müller-Taubenberger. For generating AX2 cells producing CshA-RFP, the *cshA* gene was cloned into the EcoR1 site of the 389-2 vector (C-terminal mRFPmars). AX2 cells producing CshA-RFP or RFP alone were generated by electroporation (Pang et al., 1999), and transformants were selected on plates in nutrient medium containing 10 μg/ml G418. All strains were cultured axenically in AX2 axenic medium (AX2M) (Watts and Ashworth, 1970) or in the growth media listed in Table 1 (Franke and Kessin, 1977). In all cases, cells were grown in Erlenmayer flasks under shaking at 150 rpm at 22°C on a gyratory shaker in a climatic cabinet (Kueh ner, Basel, Switzerland) as previously described (Peracino et al., 2013). Blasticidin at the concentration of 10 μg/ml was added to the Nramp1-KO mutant. Cells producing GFP- or RFP-fused proteins were cultured in the presence of 10–30 μg/ml G418.

**Table 1:** Minimal media used for cell growth.

	**Metal added (μM)**		
	**Fe**	**Zn**	**Cu**
FM^*^	100	8	0.6
M1	0	8	0.6
M1+	10–200	8	0.6
M2	100	0	0.6
M2+	100	80–800	0.6
M3	100	8	0
M3+	100	8	6–600

* *As indicated in the recipe of the FM medium defined in Franke and Kessin (1977)*.

*M1 to M3: medium composition as in FM, except for the metal indicated*.

*Escherichia coli B2* strain, *Klebsiella aerogenes*, and *Salmonella typhimurium* (ATCC number 14028) were used. Pre-cultures of bacteria were maintained in LB agar.

*Legionella pneumophila* Corby producing GFP or Ds-red Express were provided by Michael Steinert and grown on buffered charcoal yeast extract agar (BCYE; 10 g/l ACES, 10 g/l yeast extract, 2g/l activated charcoal powder, 15 g/l agar, 3.3 mM L-cysteine, 0.6 mM Fe(NO_3_)_3_ (Feeley et al., 1979) supplemented with 5 μg/ml chloramphenicol. Bacteria were incubated for 72 h at 37°C and 5% CO_2_ (Fajardo et al., 2004; Peracino et al., 2013).

### Cell growth assays

Exponentially growing AX2 cells were washed twice in 0.017 M Soerensen Na/K phosphate buffer (pH 6.0) and resuspended at a final concentration of 5 × 10^4^ cells/ml in the different media listed in Table 1. The required metal concentration was added from a stock solution of the salt dissolved in water and filtered. For iron, the stock solution was freshly prepared to avoid formation of precipitates. Cells were grown under shaking and growth was assessed daily over a period of 1 to 3 weeks, when required by subculturing the cells in the same medium shortly before they reached the stationary phase. Cell number was counted using a hemocytometer Bürker chamber. For some experiments, growth was also assessed in M2 and M3 medium with addition of 50, 100, or 500 μM zinc extracellular chelator 2-{[Bis(2-pyridinylmethyl)amino]ethylamino}benzenesulfonic acid hydrate sodium salt ZX1 (Strem Chemicals Inc., Newburyport, USA) (Pan et al., 2011) or 50, 100, or 500 μM of copper extracellular chelator trientine hydrochloride (Sigma-Aldrich, St Louis, MO, USA).

### Cell development on non-nutrient agar plates

AX2 cells were grown for 24 h in M1 ± Fe medium or for 480 h in M2 ± Zn or M3 ± Cu medium. Cells were then harvested from culture media, washed twice in Soerensen phosphate buffer and resuspended in the same buffer a concentration of 1 × 10^7^ cells/ml. Aliquots of 50 μl were plated on agar plates buffered with Soerensen phosphate buffer at a density of about 3 × 10^5^ cells per cm^2^, and incubated at 23°C (Peracino et al., 2013). Images were acquired using a Wild M3Z Stereomicroscope (Wild Heerbrugg, Switzerland) supplied with a MicrOcular 3.0 MP Electronic Eyepiece (Bresser GmbH, Rhede, Germany).

### Cell growth on bacteria

A single colony of bacteria was picked from plate and bacteria were grown in LB medium overnight with shaking. The next day, 50 μl of the bacterial culture were spread in each well of a 24-well N-agar (1 g of peptone, 1 g of glucose, 15 g of agar in 1 l of Soerensen phosphate buffer) for *E.coli B/2* and *S. typhimurium*, or SM-agar (10 g of bactopeptone, 1 g of yeast extract, 10 g of glucose, 1 g of MgSO_4_ 7(H_2_O), 2.5 g of KH_2_PO_4_, 1 g of Na_2_HPO_4_ 2(H_2_O), 18 g of agar in 1 l of water) for *K. aerogenes*, and further grown overnight at 22°C. AX2 cells were grown as previously described in the minimal media listed in Table 1. Cells were then washed twice in Soerensen phosphate buffer and serial dilutions were plated on the dry bacterial lawn, as described in Froquet et al. (2008). Pictures of the appearance and widening of growth plaques were acquired daily with a scanner.

### *Legionella pneumophila* uptake and infection assays

AX2 or Nramp1-KO cells grown in the media listed in Table 1 were washed twice in Soerensen phosphate buffer containing 50 μM CaCl2, resuspended in the corresponding culture medium without glucose containing 5 μg/ml chloramphenicol, and 1.0 × 10^5^ cells were plated in 96-well tissue culture plates. For every condition, cells were tested in triplicates for a series of time points ranging from 0 to 96 hpi. As control, AX2 cells grown in axenic medium and resuspended in low fluorescence medium (LoFlo Medium supplemented with yeast extract, Formedium, Norfolk, UK) containing 5 μg/ml chloramphenicol were used. Freshly collected *L. pneumophila* Corby producing GFP were added to *Dictyostelium* cells at a MOI (Multiplicity Of Infection) of 1:1 and immediately centrifuged at 600 g for 10 min at room temperature to synchronize infection. Plates were incubated at 25°C and samples were collected every 24 h (Hägele et al., 2000; Skriwan et al., 2002). Uninfected cells were used as a control. Flow cytometry analysis was performed according to Tiaden et al. (2013), using a CyAn ADP Analyzer (Beckman Coulter, Brea, CA, USA). Cells were identified based on forward and side scatter parameters. GFP fluorescence was measured in the FL1 channel (excitation wavelength: 488 nm; emission: 530–540 nm), and the mean fluorescence was determined for at least 10,000 cells. For some experiments, 25 μM membrane-permeable zinc chelator N,N,N′,N′-Tetrakis(2-pyridylmethyl)ethylenediamine (TPEN) (Sigma-Aldrich, St Louis, MO, USA) was added to the infection medium.

For the uptake assay, cells were grown as described and infected with *L. pneumophila* Corby producing GFP at a MOI of 10:1. After centrifugation, cells were detached and analyzed by flow cytometry at 0 and 40 min post-infection. For every time, duplicates were tested and the percentage of fluorescent cells was recorded.

Data analysis was carried out using FlowJo software (FlowJo LLC, Oregon USA).

### *In vivo* microscopy and fluorescence imaging

Cells infected with *L. pneumophila* producing GFP or Ds-red Express were plated in black 96-well tissue culture μ-plates (ibidi GmbH, Planegg/Martinsried, Germany) and incubated at 25°C. Confocal series images were taken daily on an inverted Zeiss LSM800 with AiryScan (Carl Zeiss, Inc., Oberkochen, Germany) equipped with a Plan-Apochromat 63x/1.40 DIC Oil- immersion objective. For excitation, a 488 nm Diode laser was used and its emission collected with a 495–519 nm filter for GFP and a 592–614 nm filter for RFP. Phase contrast was recorded simultaneously.

For monitoring zinc intracellular localization, cells producing Nramp1 or NrampB-RFP or AX2 cells mixed with TRITC-labeled *E. coli* (MOI 100:1) were used. Cells were washed and resuspended in Soerensen phosphate buffer and incubated for 30 min with 5 μM Zinpyr-1 fluorescent probe (Abcam, Cambridge, UK). Non-incorporated probe was removed by washing with Soerensen buffer and the cells were plated on glass coverslips. Images were acquired as previously described.

### Metal analysis by inductively coupled plasma mass spectrometry (ICP-MS)

AX2 cells were grown for 24 h in M1 ± Fe medium or for 480 h in M2 ± Zn or M3 ± Cu media. A total of 10^8^ cells were washed five times in Soerensen phosphate buffer and pelleted in Eppendorf tubes. Digestion was performed by adding 1 ml of concentrated HNO_3_ (70%) to each sample. After complete dissolution, samples were further digested by applying microwave heating (Milestone MicroSYNTH, Microwave labstation equipped with an optical fiber temperature control and HPR-1000/6M six position high-pressure reactor, Bergamo, Italy). After digestion, the volume of each sample was brought to 3 ml with ultrapure water, filtered with 0.45 mm filter and analyzed by ICP-MS, using a Thermo Scientific ELEMENT 2 ICP-MS (Finnigan, Rodano, Italy) (Fiorito et al., 2012). The same analysis was also performed on 800 μl of M2 or M3 medium. The quantification was obtained through a calibration curve measured by using six Fe/Cu/Zn absorption standard solutions (Sigma-Aldrich) in the range 0.0025–0.3 μg/ml. Samples with metals concentrations higher than the upper limit were diluted opportunely. Sample digestion and metal quantifications were carried out by the facility of the Molecular Imaging Center (Department of Chemistry, University of Torino, Italy).

## Results

### Effects of transition metal depletion or overloading on growth of wild type AX2 cells

**Figure 1:**
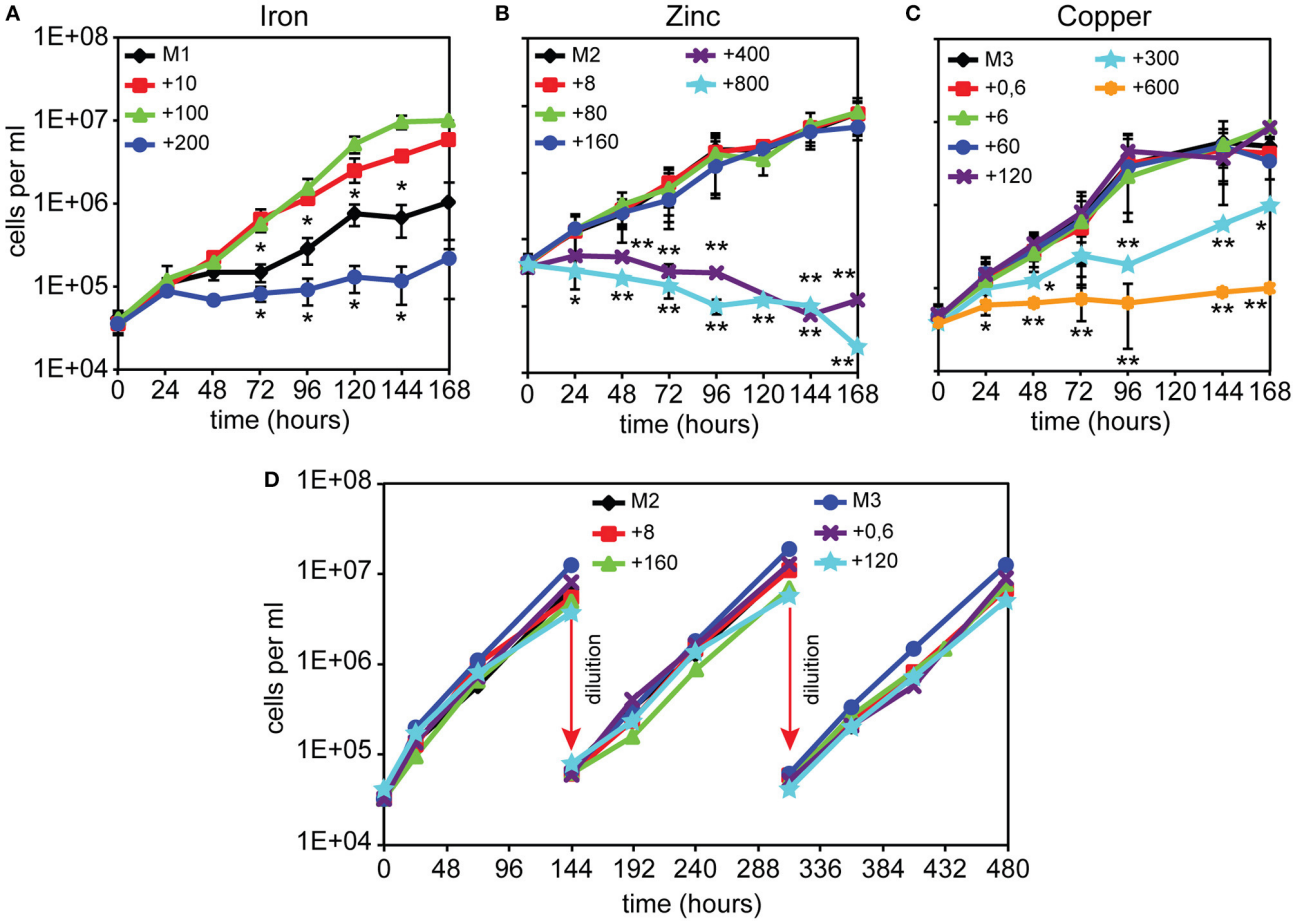
*Dictyostelium* cell growth in minimal medium with or without FeCl_3_, ZnSO_4_, or CuSO_4_ supplementation. **(A)** In the absence of iron, AX2 cells were able to replicate for 4–5 generations, albeit very slowly, before reaching a plateau. Cells grew well with 10 or 100 μM iron, whereas with 200 μM iron growth was impaired. Modified from Peracino et al. (2013). **(B–D)** Both zinc and copper deprivation did not affect AX2 cell replication. Only with 300 μM copper, growth was severely reduced and stopped completely at twice the concentration, while 400 and 800 μM zinc caused cell death. Exponentially growing AX2 cells were diluted in minimal medium with or without the indicated amounts of iron chloride **(A)**, zinc sulfate **(B)**, or copper sulfate **(C)**, incubated under shaking and counted for growth at the indicated times. **(D)** Cells were grown in the indicated amounts of zinc or copper and, before entering the stationary phase, subcultured by dilution for two additional weeks (arrows). For simplicity, only a few counts are shown. Mean values of at least three experiments with error bars (±SD) are shown. The asterisks denote significant difference compared with cells growth in minimal medium containing normal metal concentration (100 μM for iron, 8 μM for zinc, and 0.6 μM for copper). Two-tailed *t*-test assuming unequal variance: ^*^P < 0.05, ^**^P < 0.01. **(D)** Mean values of at least two experiments are shown. The numbers indicate metal concentration in μM. All media used are described in Table 1.

We first investigated how alterations in the availability of iron, copper, or zinc would affect *Dictyostelium* cell growth. This was achieved by evaluating AX2 wild type cell growth in a defined FM minimal medium (Franke and Kessin, 1977), in which the concentrations of single metals was modified. The FM minimal medium contains 100 μM FeCl_3_, 8 μM ZnSO_4_, and 0.6 μM CuSO_4_ (Franke and Kessin, 1977). We omitted or added to the original recipe one of these three metals at varying concentrations, as summarized in Table 1. AX2 cells exponentially growing in AX2 medium (AX2M) were diluted in these minimal media to an initial concentration of 5 × 10^4^ cells/ml, and growth was evaluated under shaking for 1 to 3 weeks. As previously reported, cell growth is optimal at an iron concentration of 100 μM, but inhibited after a few generations if iron is omitted or the amount increased to 200 μM (Figure 1A and Peracino et al., 2010). In sharp contrast, cell growth up to 10^7^/ml was unaffected in M2 or M3 medium or with up to 20- or 200-fold excess of zinc or copper, respectively, compared to control FM (Figures 1B,C). Higher concentrations of copper led to a delay in the rate of cell duplication and total inhibition with 1,000-fold excess (Figure 1C). In the case of zinc, a toxic effect was observed with 50 or 100-fold excess (Figure 1B).

To assess whether cell growth would be affected by prolonged incubation in medium without added zinc or copper or with high, but non-toxic, concentrations of zinc or copper, namely 20- or 200-fold excess, respectively, cells were re-diluted in the same medium, shortly before reaching the stationary phase, and cell growth measured over 480 h. As shown in Figure 1D, no significant changes in the rate of cell doubling time was observed. Thus, *Dictyostelium* cells appear to be very efficient in controlling high concentrations of copper or zinc. More surprising is that their growth rate is unaffected by depletion of either metal. A preliminary analysis of the metal proteome, by using a modified metal predator as bioinformatic tool (Valasatava et al., 2016), shows that the *Dictyostelium* genome encodes 840 zinc- and 250 iron-binding proteins, corresponding to 6.6 and 2%, respectively, of the entire proteome. There are fewer copper-binding proteins: about 40. Especially for zinc, one would expect that zinc shortage in the medium would have detrimental effects for the activity of the zinc-binding proteins, many of which are involved in basic cellular processes, such as transcription, translation, cell replication, and protein turnover. We thus tested whether incubation for 480 h, i.e., about 24 generations, in M2 or M3 medium led to a shrinkage in cell volume, but no significant changes in size with control cells were found (M2: 4.8 ± 0.9 μl; M3: 5.2 ± 1.5 μl; FM: 4.7 ± 1.8 μl, in all cases per 10^7^ cells). Addition to M2 or M3 medium of extracellular zinc chelator ZX1 or copper chelator trientine hydrochloride at concentrations varying between 50 and 500 μM also failed to affect cell growth.

### Copper and zinc concentration in cells growing in medium depleted or overloaded with metals

Presuming there are no other sources of these metals in the medium, at any cell doubling the intracellular amount of iron, zinc, or copper should halve. Initially, cells contain the metals engulfed with the axenic medium and might undergo some duplication rounds, as observed for iron (Figure 1A), but this does not explain the ability of the cells to sustain more than 20 generations in the absence of external zinc or copper (Figures 1B,C). However, even in minimal medium without added metals, metal contamination cannot be excluded, particularly due to amino acids. Indeed most amino acids contain up to 0.5–5 ppm iron, zinc or copper, based on manufacturer sheets. In addition, metals can leach from the glassware and, depending on their composition, from some plastics used for cell culture and treatment (Kay, 2004).

To get an unbiased measure of the amount of zinc or copper in medium and in cells, we measured their concentrations by using Inductively Coupled Plasma Mass Spectrometry (ICP-MS). In the nominally metal-free M2 and M3 medium, 0.58 and 0.028 μM zinc or copper, respectively, were detected, amounting to 7.26 and 4.67% the concentration of zinc or copper, respectively, present in the standard FM medium (Table 2). When cells subcultured for 3 weeks in M2 or M3 medium were analyzed by ICP-MS, we found that 10^7^ cells still contained about 84 or 3.1 ng of zinc or copper, respectively, i.e., about 87 and 51% of the amount present in cells growing in standard minimal medium or AX2 medium. In cells incubated with a 20 or 200-fold excess of zinc or copper, respectively, the cellular concentration increased of only 2.7- and 2.4-fold, respectively, compared to FM or AX2 medium (Table 3). Normalized for cell volume, the intracellular copper concentration in *Dictyostelium* is 6 ± 0.7 and 30.6 ± 0.3 μM for cells grown in, respectively, nominally copper-free medium or medium containing 120 μM Cu. The intracellular zinc concentration amounts to 243 ± 12.3 to 912 ± 37.7 μM in cells grown in, respectively, nominally zinc-free medium or in presence of 160 μM zinc. The intracellular values are, at least for zinc, comparable to those found in many eukaryotic cells (Krezel and Maret, 2006; Colvin et al., 2008; Vignesh et al., 2013; Kambe et al., 2015). While incubation in M2 or M3 medium slightly affected the intracellular amount of zinc or copper after 3 weeks of culture, just 24 h of growth in M1 medium (corresponding to 1 cell doubling) were sufficient to halve the iron cellular content (Table 3). ICP-MS analysis in cells incubated with excess of iron could not be performed for the persistence in the sample of iron precipitates that would bias the measure.

**Table 2:** ICP-MS analysis of media.

	**[Me] (μM)**	
	**Zn**	**Cu**
FM^*^	8	0.6
M2	0.581	
M3		0.028

* *The concentration of metals added to the FM medium are reported. The nominally metal-free media M2 and M3 were subjected to ICP-MS analysis as described in Material and Methods*.

**Table 3:** Cellular metal content (ICP-MS).

**Growth in:**	**Me/10**^**7**^**cells (ng)**		
	**Fe**	**Zn**	**Cu**
AX2M	31.1 ± 19.1	93.5 ± 20.1	6 ± 0.1
FM	68.9 ± 0.3	96.2 ± 11.2	6 ± 0.4
M1	17.3 ± 0.2		
M2		84 ± 4.2	
M2+160		253.4 ± 10.5	
M3			3.1 ± 0.4
M3+120			14.6 ± 0.1

*Total concentration of Zn or Cu measured by ICP-MS in lysates from cells grown for 24 h in M1 ± Fe or for 3 weeks in M2 ± Zn or M3 ± Cu. Cells growing in AX2M and FM were tested after 24 h for Fe, and 3 weeks for Zn and Cu. Data are means ± SD of one experiment in duplicate. The cell lysate concentrations are based on 10^8^ cells in ~130 μl*.

These results indicate that, unlike iron, *Dictyostelium* cells are extremely efficient in acquiring and storing copper and zinc, maintaining intracellular levels sufficient to support growth, even when the concentration of these metals, particularly zinc, in the medium is strongly reduced. Similarly, their high tolerance toward both zinc and copper toxicity seems to derive from their high efficiency in extruding excessive amounts of these metals from the cell.

### Effects of iron, zinc, and copper shortage or overload on growth on bacteria and development

**Figure 2:**
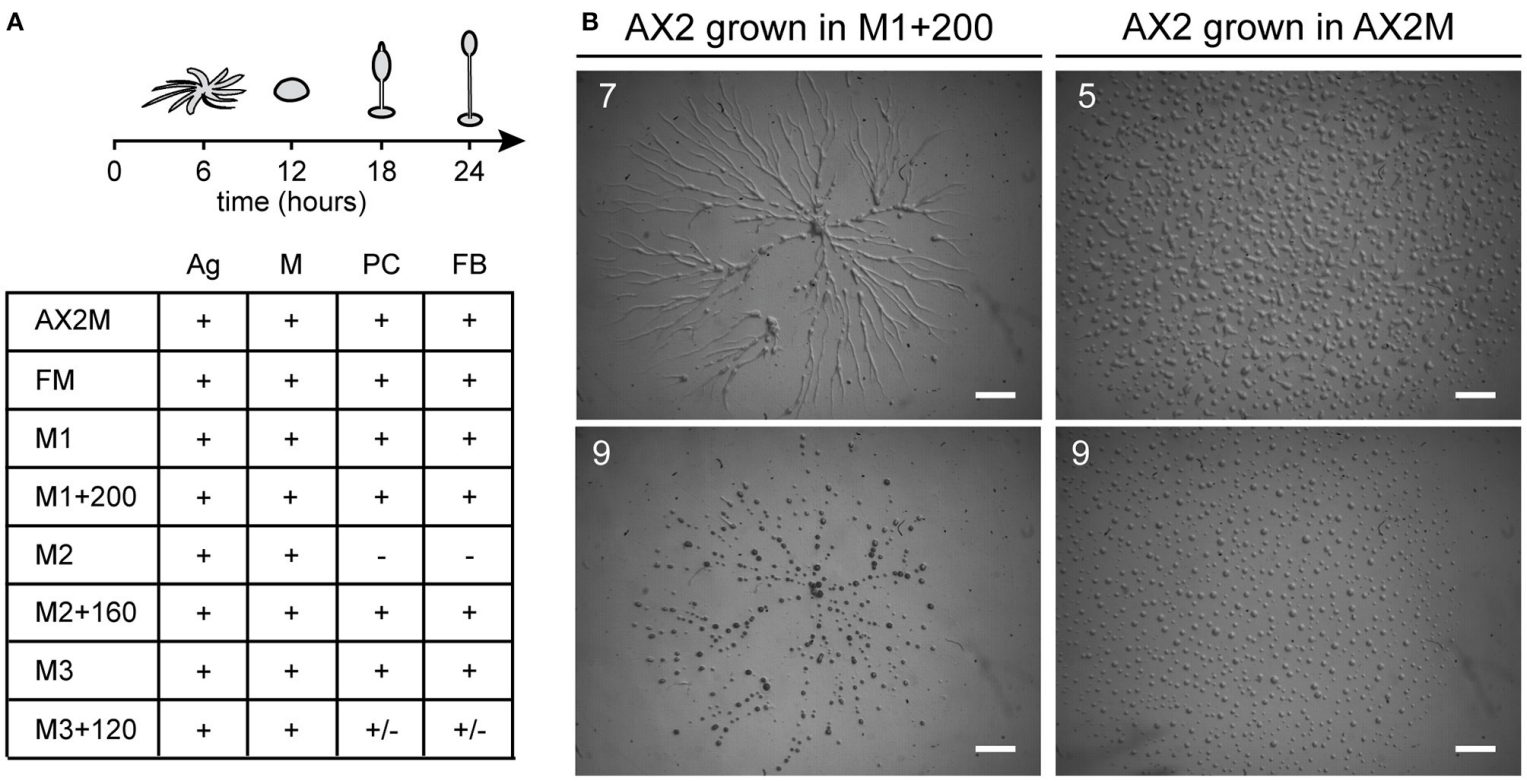
Effects of metal deficiency or overload on *Dictyostelium* development. AX2 cells were pre-incubated for 24 h in M1 ± Fe, or for 3 weeks in M2 ± Zn, or M3 ± Cu. Thereafter, cells were washed in Soerensen phosphate buffer, plated on non-nutrient phosphate agar and monitored over time in a stereomicroscope. **(A)** (Top) Schematic representation of development with timing: stream formation (aggregation, Ag), mounds (M), pre-culminants (PC), and mature fruiting bodies (FB). (Bottom) Developmental stages reached by cells grown in the indicated medium. Symbols indicate: “+” stage reached at the indicated time; “–” developmental stage not reached; “+*/–*” stage reached, but with alterations in time or morphology. Results from three different experiments. **(B)** AX2 cells grown in M1+200 for 24 h, in contrast to cells grown in AX2 medium (on the right), formed very elongated streams that fragmented in multiple mounds. The numbers indicate hours from the beginning of cell starvation. Scale bar: 1 mm.

Cells previously grown under deficiency or overloading of iron, zinc, or copper were tested for development on agar. For iron, we employed the same conditions used for the growth assay (Figure 1A): cells were incubated in nominally iron-free M1 medium or with iron supplementation for 24 h, washed in Soerensen phosphate buffer and then analyzed for development. Cells grown in M1 developed normally. Cells grown in M1 plus 200 μM iron developed with normal timing (Figure 2A), but formed very large streams, which underwent fragmentation into multiple mounds that developed to fruiting bodies (Figure 2B). In the case of copper or zinc overloading, we tested concentrations that produced no toxic effects on cell growth, and the cells were grown for 3 weeks (Figure 1D), before being washed and assayed for development. Cells were able to develop and form fruiting bodies similarly to control cells, except for cells grown in M2 medium which failed to complete development and were arrested at the mound stage (Figure 2A). Cells grown in a 200-fold excess of copper formed loose mounds, which elongated to small fruiting bodies, though many cells failed to aggregate (Figure 2A).

To assess whether previous growth under metal deficiency or overloading affected phagocytosis and growth on non-pathogenic bacteria, cells grown as just described were also plated on agar with *E. coli B/2, K. aerogenes*, or *S. thyphimurium*, and the increase in size of the colonies scored every 24 h. The growth rate was similar for cells grown under iron, zinc, or copper deficiency or overload on all three bacterial lawns, indicating that phagocytosis and bacterial digestion are unaffected by the previous growth conditions of the cells. In all cases, fruiting bodies were also formed in the middle of the plaque (Figure S1).

### Dynamics of *Legionella* infection analyzed by flow cytometry and live-cell imaging

**Figure 3:**
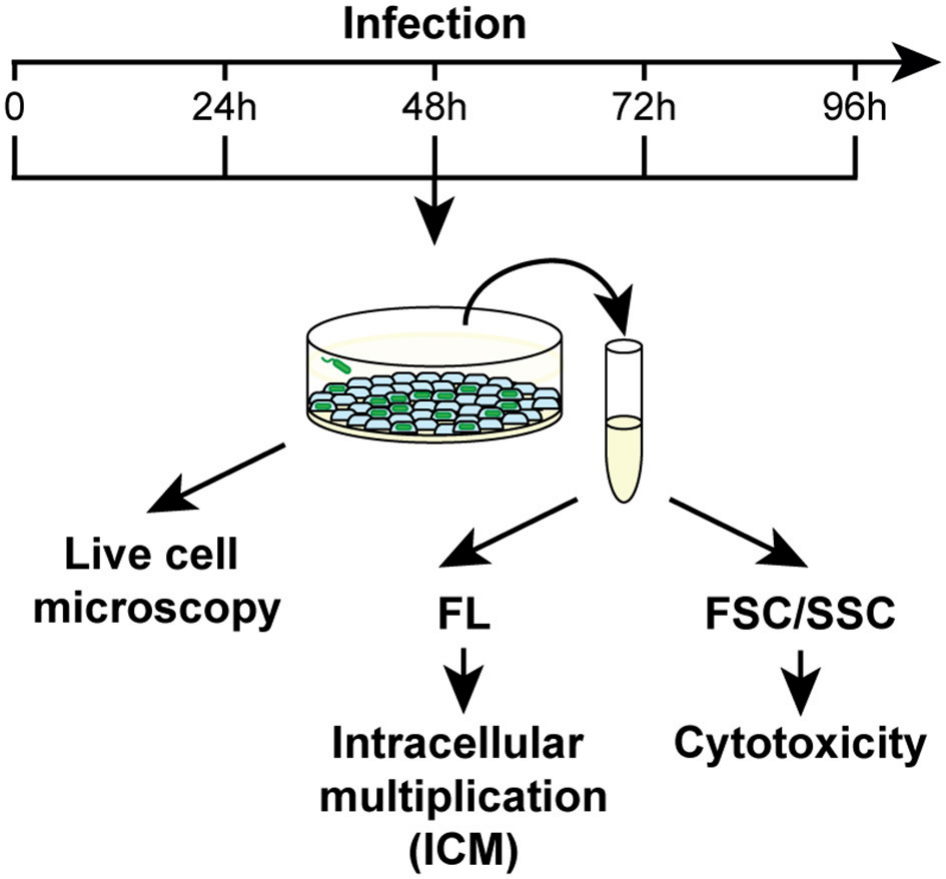
Work flow for *Legionella* infection assays. *Dictyostelium* cells were infected with GFP producing *L. pneumophila* in 96-well plates at MOI 1:1 and analyzed daily by confocal microscopy or by flow cytometry. For the latter, two different parameters were acquired: the increase of mean fluorescence over time as a measure of the intracellular replication of bacteria, and changes in the FSC/SSC pattern as indicators of cytotoxicity. Concomitantly, cells were monitored by confocal microscopy. Abbreviations are as follows: *FL* fluorescence, *FSC/SSC* forward/sideward scatter channel.

**Figure 4:**
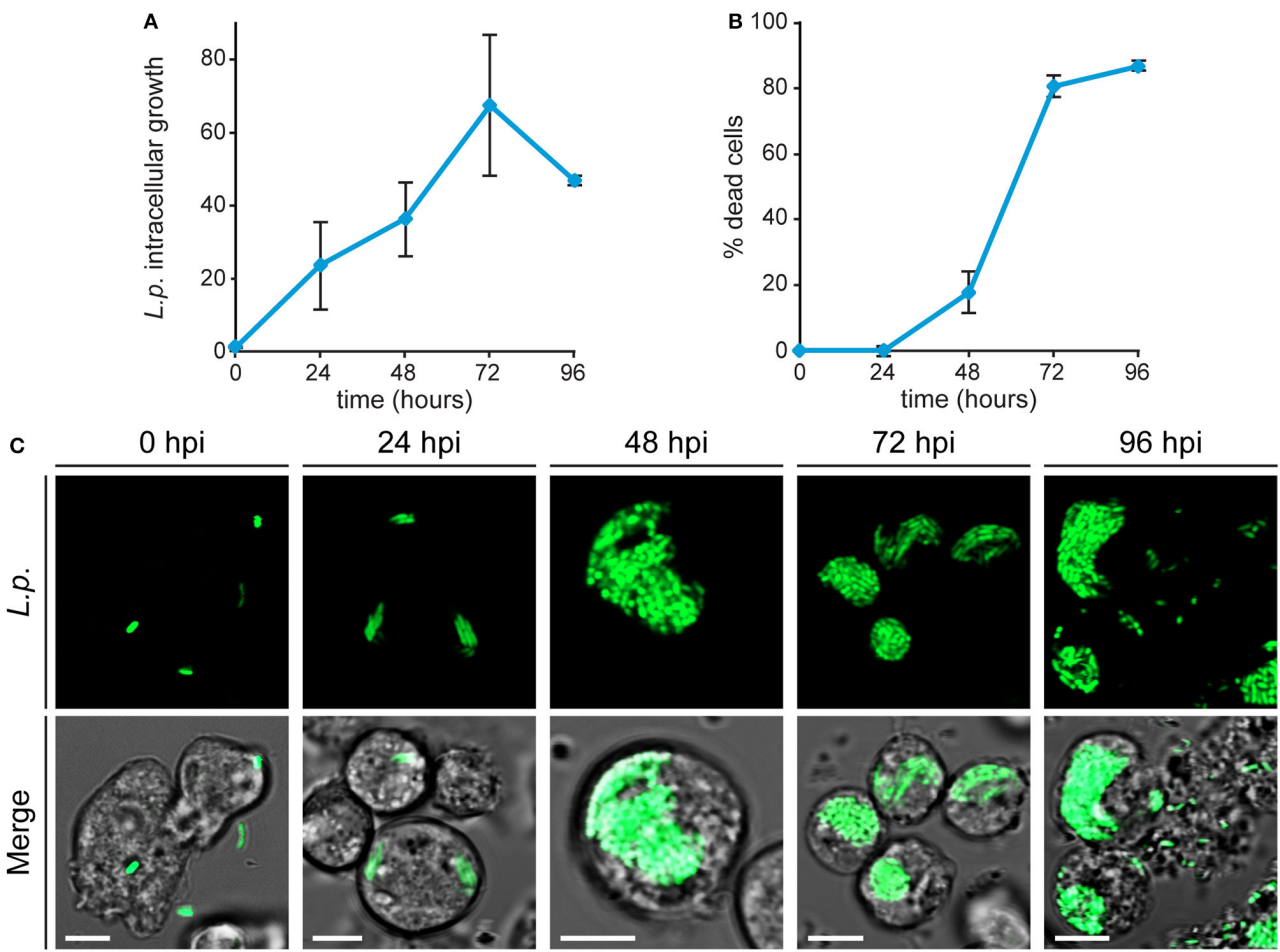
*Legionella* infection in *Dictyostelium* cells. **(A,B)** AX2 cells growing axenically were infected with GFP-producing *L. pneumophila* and monitored by flow cytometry. The intracellular growth of *Legionella* was assessed by measuring the mean fluorescence of GFP-producing *L. pneumophila* within AX2 cells and *L. pneumophila*-triggered cytotoxicity by determining the percentage of dead cells. For ICG, the trend in different experiments was similar, although the absolute values varied strongly between replicates, due to changes in cell autofluorescence. Thus, *Legionella* replication was expressed as mean fold-increase in fluorescence intensity relative to the autofluorescence of the corresponding non-infected cells. All measurements are mean values of seven different experiments with error bars (±SD). **(C)** Confocal fluorescence images of infected living cells at different times post-infection. (Top) GFP-producing *Legionella* and (Bottom) merge with corresponding phase-contrast. In intact cells, the proliferating bacteria are packed together, except for extracellular bacteria in lysed cells (96 hpi). Scale bars: 5 μm.

The effects of metal deficiency or overload on *Dictyostelium* resistance to pathogenic bacteria, was studied with *Legionella pneumophila*. *L. pneumophila* infection was monitored, as schematized in Figure 3, by confocal microscopy and by measuring in flow cytometry cellular fluorescence due to intracellular proliferation of GFP-producing bacteria, and cytotoxicity as changes in cellular physical parameters. In control cells grown in AX2 medium and incubated with *Legionella*, the intracellular fluorescence increased over time, reaching a peak 72 h post-infection (hpi), and decreasing thereafter (Figure 4A). Concomitantly, the percentage of dead cells (identified as a smaller and granulous population) incremented, exceeding 80% at 72–96 hpi (Figure 4B). Indeed, as observed by confocal microscopy, *Legionella* started to replicate inside the infected cells soon after its entry, with about 5–15 bacteria/cell 24 hpi, continuing to proliferate until filling almost completely the cell. The host cells lost the amoeboid form, rounding up and detaching from the bottom of the well by around 24 hpi. From 72 hpi, but strongly at 96 hpi, we noticed the presence of cellular debris and an increase in the number of extracellular bacteria, indicating cell lysis (Figure 4C). Under the conditions used, *Legionella* cannot grow extracellularly, thus an increase in the number of extracellular bacteria can only be the result of leakage from lysed cells. This explains also the reduction of cellular mean fluorescence measured at 96 hpi (Figure 4A).

**Figure 5:**
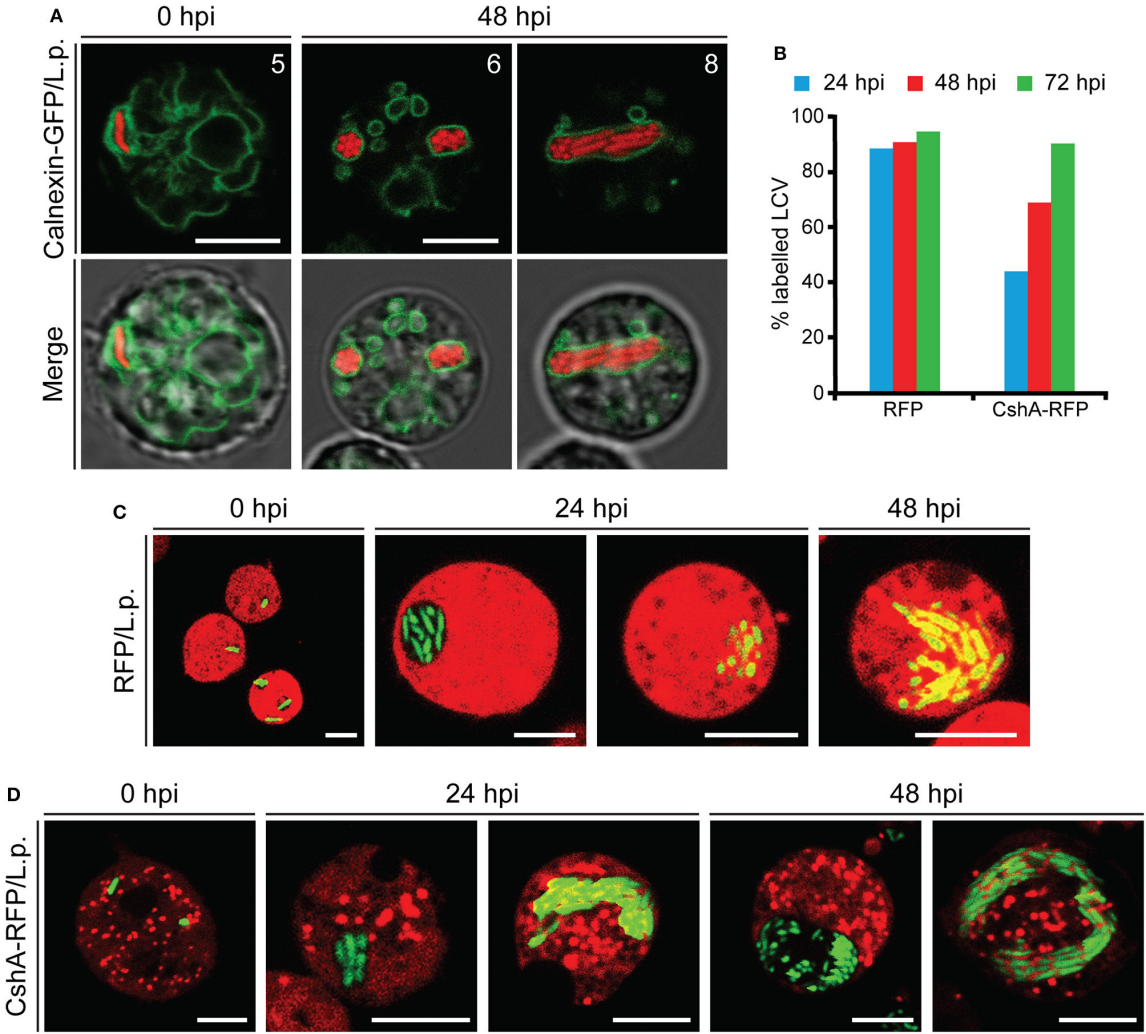
Characterization of the LCV membrane during *Legionella* infection. **(A)** Confocal images of living AX2 cells producing Calnexin-GFP and infected with RFP-producing *L. pneumophila*. The double exposure (red and green fluorescence) shows the presence of a membrane surrounding the replicating bacteria even at 48 hpi. The numbers indicate distance from the bottom surface of the cell (μm). **(B–D)** Co-localization of RFP or CshA-RFP with the LCV. AX2 cells expressing free RFP or CshA-RFP were infected with GFP-producing *L. pneumophila* and confocal images acquired every 24 h post-infection **(C,D)**. The small organelles labeled with CshA-RFP in **(D)** are peroxisomes. **(B)** The number of LCVs labeled with the cytosolic fluorescent proteins was quantified analyzing at least 30 cells for each time point and expressed as percentage of the total. For all confocal images, red and green fluorescence merge is shown. The diffusion of free-RFP or CshA-RFP fluorescence inside the LCV allows evaluating the integrity of the LCV membrane. Scale bars: 5 μm.

After its entry, *L. pneumophila* forms a replication-permissive compartment inside the host cell, known as the *Legionella*-containing vacuole (LCV) (Horwitz, 1983; Lu and Clarke, 2005; Steiner et al., 2017). We observed that, even at later stages of infection, the bacteria appeared to be packed together, as if contained in a cellular compartment (Figure 4C and Movie S1). We infected *Dictyostelium* cells producing calnexin-GFP with a RFP-producing *Legionella* strain. Calnexin is an ER-specific protein that was previously demonstrated to be recruited to LCVs (Fajardo et al., 2004; Ragaz et al., 2008), where it remains for at least 8–14 hpi (Urwyler et al., 2009; Weber et al., 2014). Using this marker, we evidenced the presence of a membrane surrounding the large mass of replicating bacteria even at 48 hpi (Figure 5A), indicating that the LCV continues to tightly interact with the ER also at advanced stages of infection. The membrane integrity of the LCV was then evaluated by infecting cells producing free RFP or CshA-RFP. The citrate synthase CshA is a cytosolic protein, composed of 492 amino acid residues and containing a peroxisomal targeting PTS2 sequence, which by interacting with Pex7 leads to its transport to peroxisomes (Huang et al., 2004). In CshA-RFP producing cells peroxisomes are labeled, in addition to a diffused labeling of the cytosol, due to the synthesized, but not yet transported, protein. Cellular compartments whose membrane is not permeable appear as “black holes” in RFP or CshA-RFP producing cells. At 24 hpi the replicating legionellae were clearly contained within a RFP or CshA-RFP impermeable membrane (Figures 5C,D). In some cells the RFP or CshA-RFP labeling diffused between bacteria, suggesting that the LCV membrane had become permeable to the fluorescent proteins (Figures 5C,D). As infection progressed, the proportion of cells showing the latter condition increased. Since RFP is smaller than CshA-RFP (27 kDa vs. at least 75 kDa for the CshA-RFP non-complexed with Pex7), we calculated the percentage of LCVs showing co-localization of with either RFP or CshA-RFP at each time point. As shown in Figure 5B, at 24 hpi only 44% of LCVs were positive for CshA-RFP against 88% for RFP, with a gradual increase to 94 or 90% at 72 hpi for RFP or CshA-RFP, respectively. It is worth mentioning that no peroxisomes were found mixed with legionellae within the LCV, even at 72 hpi, but only free RFP or CshA-RFP.

From these data we conclude that the LCV continues to be surrounded by a membrane even at later stages of infection, with the membrane becoming gradually permeable to proteins of increasing molecular size between 24 and 72 h after its formation, with no egression of bacteria in the cytosol, but dispersal into the extracellular milieu following cell lysis.

### Legionella uptake and infection in AX2 or Nramp1-KO cells grown in minimal medium with or without iron supplementation

**Figure 6:**
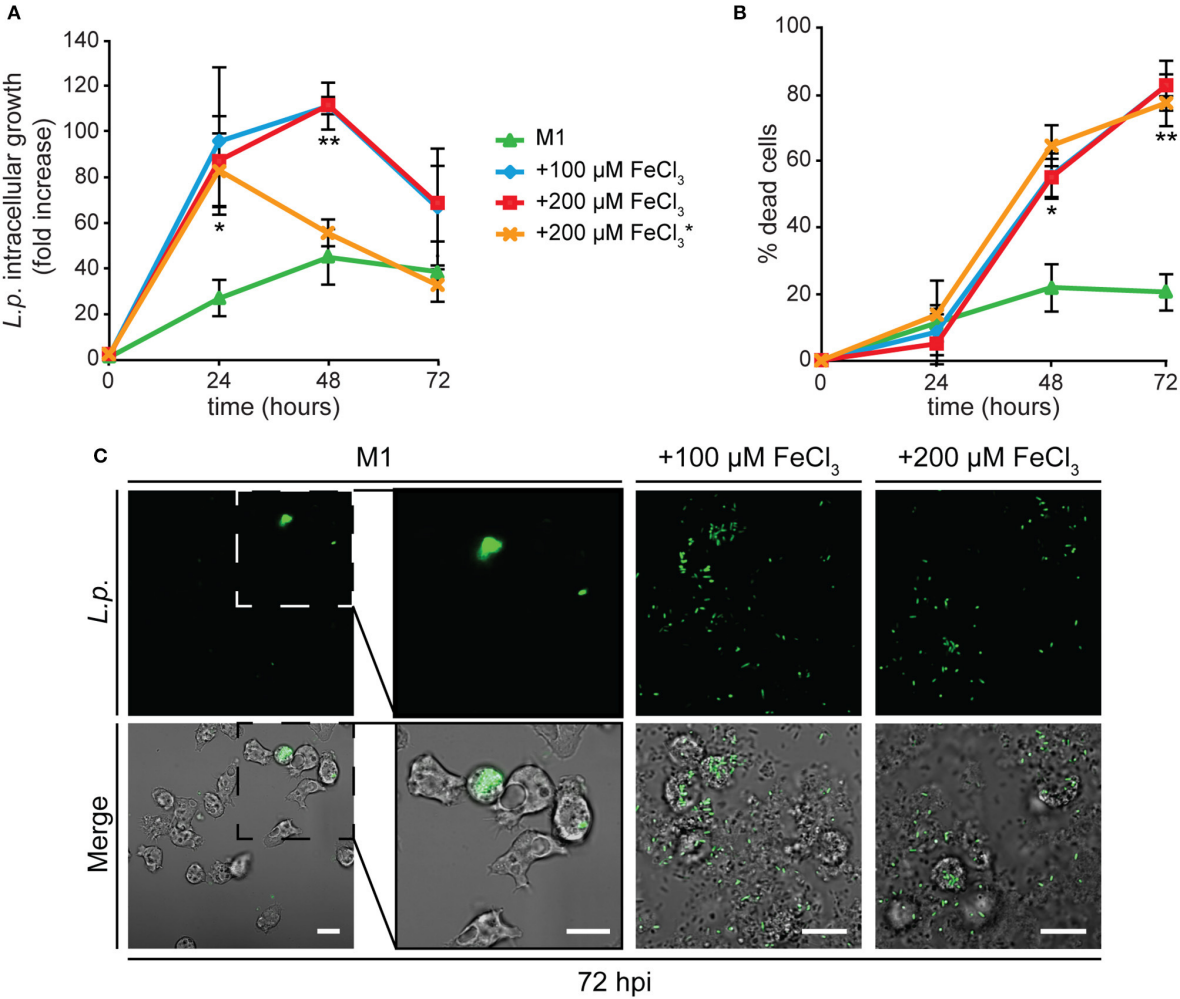
Alterations in cellular iron content affect *Legionella* infection in *Dictyostelium*. **(A,B)** AX2 cells were grown for 24 h in M1 ± Fe and infected with GFP-producing *L. pneumophila*. In some experiments with cells grown in M1+200 μM iron, iron at the same concentration was added to the medium during infection (^*^). The intracellular growth (ICG) of *Legionella* **(A)** and cytotoxicity **(B)** were measured by flow cytometry as in Figure 4. AX2 cell growth in M1 results in strongly reduced *Legionella* growth and cytotoxicity, whereas growth in iron-rich media stimulates both *Legionella* intracellular growth and induced cytotoxicity. The presence of iron in the medium during infection induces earlier bacterial leakage from infected cells, shifting the fluorescence peak from 48 to 24 hpi. Mean values of two to four experiments in triplicate with error bars (±SD). ^*^P < 0.05 or ^**^P < 0.01 (two-tailed Student's *t*-test, assuming unequal variance) vs. the condition of growth in M1. **(C)** Confocal images of infected cells at 72 hpi. Green fluorescence and merge with corresponding phase-contrast are shown. In M1 only few cells are infected with *Legionella* after 72 h and no bacteria are visible in the extracellular medium. On the contrary, many extracellular bacteria and lysed cells are visible when cells were grown in the presence of iron. Scale bars: 5 μm.

We followed *Legionella* infection in AX2 cells grown for 24 h in minimal medium containing 0, 100, or 200 μM FeCl_3_. In cells deprived of iron during growth, *Legionella* intracellular replication was strongly reduced (Figure 6A) and the percentage of dead cells did not increase significantly over time (Figure 6B). These data were confirmed by live microscopy: at 72 hpi, only a very small number of cells were infected with bacteria (Figure 6C).

When cells previously grown in the presence of iron were tested, *Legionella* intracellular growth was significantly higher compared to the condition of iron shortage, with the number of intracellular bacteria rapidly increasing during the first 48 hpi, and starting to decrease concomitantly with a boost in the percentage of dead cells (Figures 6A,B). Indeed, by confocal microscopy we observed already at 48 hpi and strongly at 72 hpi an increase in the number of extracellular bacteria and of lysed cells (Figure 6C). No differences were detected when the amount of iron in the growth medium was doubled to 200 μM (Figures 6A,B). Despite the inhibition of growth induced by the latter concentration of iron (Figure 1A), no increase in the percentage of dead cells was observed in the uninfected control (4.8% of dead cells compared to 77.3% in infected cells at 72 hpi). Therefore, the cytotoxic effect observed in infected cells is not due to the metal, but to the rapid proliferation of the bacteria. Adding iron during the infection assay to cells previously grown in the presence of iron facilitated *Legionella* intracellular growth, with a maximal value already reached at 24 hpi (Figure 6A).

**Figure 7:**
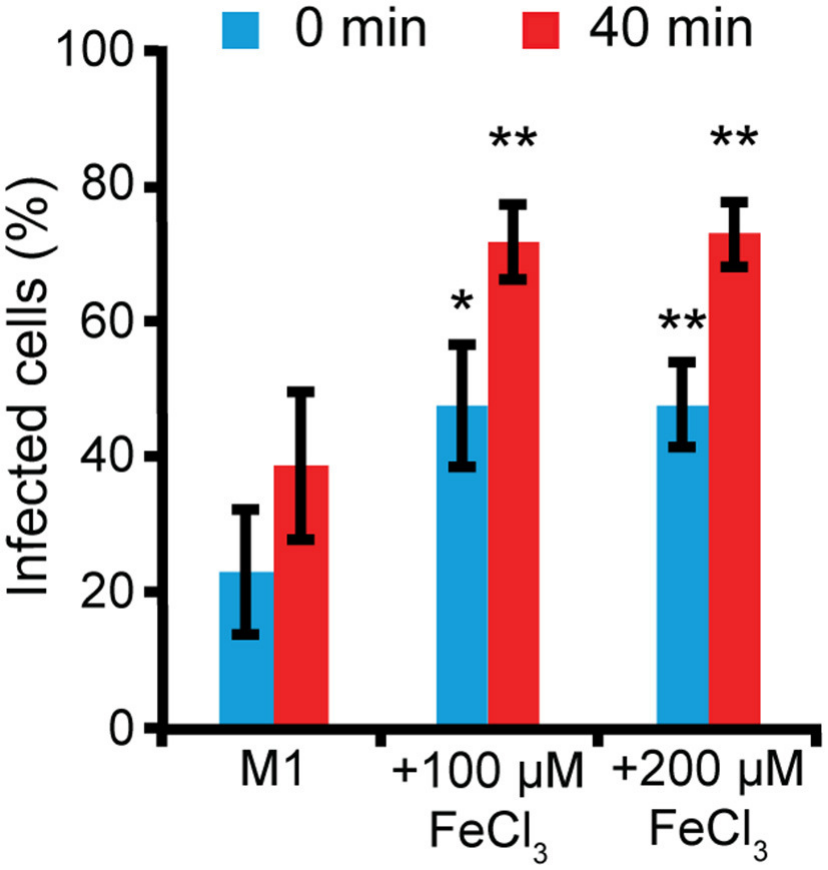
Iron deficiency or overload affects *L. pneumophila* uptake. AX2 cells grown for 24 h in M1 medium ± Fe were infected with GFP-producing *L. pneumophila* at MOI 10:1 and analyzed by flow cytometry. Bacteria uptake was measured as the percentage of *Dictyostelium* cells harboring GFP-positive *L. pneumophila* at 0 and 40 min post-infection. In the nominally iron-free M1 medium, bacteria uptake was significantly reduced compared to cells grown in the presence of iron. Mean values of three different experiments with error bars (±SD) are shown. ^*^P < 0.05 or ^**^P < 0.01 (two-tailed Student's *t*-test, assuming unequal variance) vs. the corresponding time of the condition in M1.

We also tested whether iron could affect *Legionella* uptake. AX2 cells were grown for 24 h in M1 ± Fe, as previously described. Thereafter, they were washed, resuspended in nominally iron-free medium, co-centrifuged with GFP-producing *Legionella* (MOI 10:1) and incubated for 40 min at 25°C. Uptake was measured as increase of the percentage of fluorescent (i.e., harboring bacteria) cells. When cells grown in M1 were assayed, only about 23% of them were fluorescent, rising to 38% 40 min post-infection (Figure 7). In cells grown in M1 plus 100 or 200 μM iron, the percentage of fluorescent cells was already significantly higher at time 0 (47–50%), and further increased over 66% after 40 min, with no differences between 100 and 200 μM Fe (Figure 7). It is worth mentioning that though the number of infected cells increased, the mean number of *Legionella* per cell did not increase significantly, as confirmed by confocal microscopy. Thus, growing cells in the presence or absence of iron affects both uptake and infection of *Legionella*.

**Figure 8:**
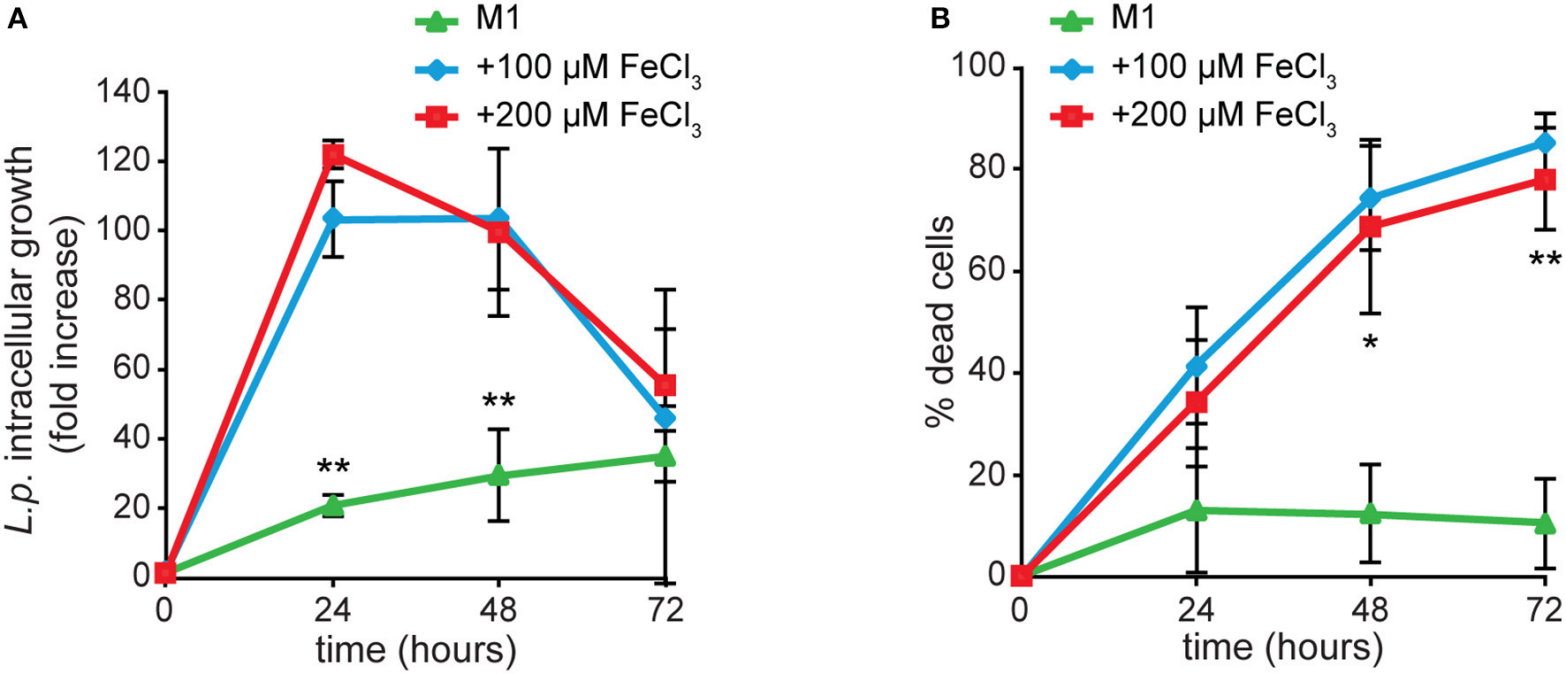
Iron effects on *L. pneumophila* intracellular replication and cytotoxicity in Nramp1-KO mutant. Infection was evaluated in Nramp1-KO cells as previously described for AX2 cells in Figure 5, in the presence of iron in the infection medium at the same concentrations used for cell growth. Flow cytometry analysis of ICM **(A)** and cytotoxicity **(B)** are shown. In cells lacking Nramp1, growth in M1 affected bacterial replication and cytotoxicity similarly to AX2, whereas growth in the presence of iron stimulated *Legionella* infection to a higher degree than in AX2 (see Figure 5). Mean values of at least two experiments in triplicate with error bars (±SD). ^*^P < 0.05 or ^**^P < 0.01 (two-tailed Student's *t*-test, assuming unequal variance) vs. the condition of 100 μM FeCl_3_.

We previously showed that *Dictyostelium* Nramp1 is rapidly recruited from the Golgi to phagosomes and macropinosomes. Here it mediates iron efflux to the cytosol and, by doing so, it confers resistance to infection by intracellular pathogens, such as *Legionella* and *Mycobacterium avium* (Peracino et al., 2006, 2013; Buracco et al., 2015). Similarly to AX2 cells, when Nramp1-KO cells were grown for 24 h in M1 medium, infection was significantly reduced in both parameters, *Legionella* intracellular replication and bacteria-triggered cytotoxicity (Figure 8). In cells grown in the presence of iron and infected in a medium containing the same concentration of the metal, *Legionella* intracellular growth reached again a peak at 24 hpi and then started to decrease over time (Figure 8A). At the same time, the percentage of dead cells increased steadily, exceeding 80% at 72 hpi (Figure 8B). Thus, iron deficiency or overload has similar effects on *Legionella* infection in cells lacking Nramp1 and parental cells, though in the Nramp1-KO mutant intracellular growth of *Legionella* was more rapid, consistent with its increased susceptibility to infection (Peracino et al., 2006).

### Effects of altering zinc or copper availability on *Legionella* infection

**Figure 9:**
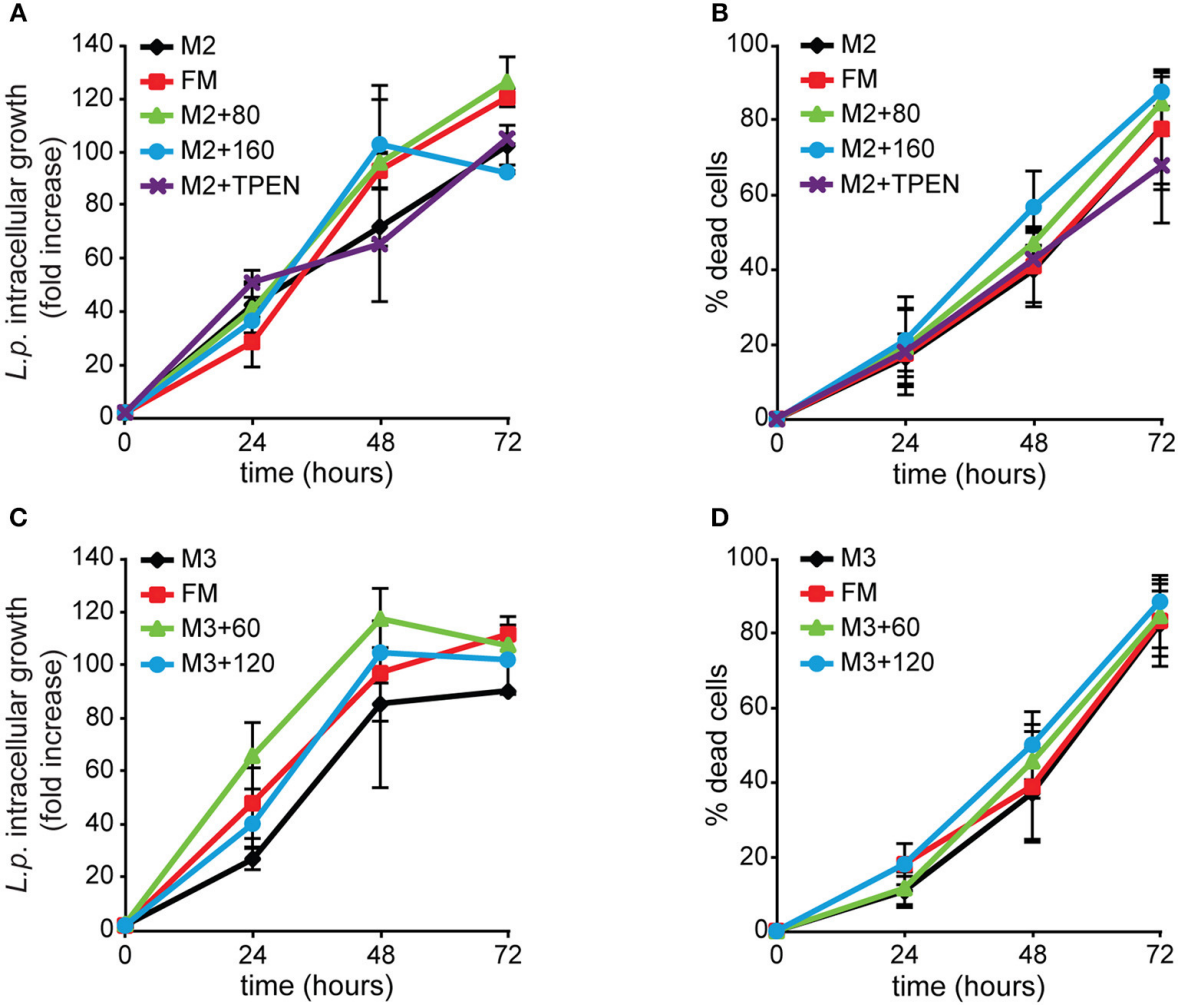
Alterations of zinc or copper availability do not affect *Legionella* infection in *Dictyostelium* cells. AX2 cells were grown for 3 weeks (24 generations) in **(A,B)** M2 ± Zn or **(C,D)** M3 ± Cu medium and then infected with GFP-producing *L. pneumophila*. ICG and cytotoxicity were evaluated as previously described in Figure 4, with the first expressed as mean fold-increase in fluorescence intensity relative to the autofluorescence of the corresponding non-infected cells. Omitting or adding zinc or copper in cell growth medium did not significantly affect *Legionella* intracellular growth and induced cytotoxicity compared to FM medium. In the case of zinc, adding the intracellular chelator TPEN during infection failed to have any effect. Infection in cells grown in M3 + 6 μM Cu was comparable to the other conditions and, for simplicity, it was removed from the graphics. Mean values of three different experiments with error bars (±SD). **(C)** *P* = 0.054, at 24 hpi for M3 compared to M3 + 60 μM. The numbers indicate metal and chelator concentration in μM.

We tested whether zinc or copper exert similar effects as iron. AX2 cells grown for 168 h in M2 ± Zn or M3 ± Cu were infected and analyzed by flow cytometry. Under all conditions tested, neither bacterial intracellular proliferation nor cytotoxicity were significantly altered compared to the control incubated in standard FM medium (Figure 9). As shown in Table 3, omitting zinc or copper in the culture medium affects only minimally the cellular concentration of these metals, particularly for zinc, compared to the standard FM medium, with no inhibitory effect on cell growth, though in the case of zinc postaggregative development is blocked. In some experiments the intracellular zinc chelator TPEN was added during *Legionella* infection to cells previously grown in M2 medium, but no effects on *Legionella* growth or cytotoxicity were observed compared to the M2 medium (Figure 9). The absence of significant differences in *Legionella* infection between cells grown in nominally-free metal medium, supplemented in the case of zinc with TPEN during infection, or at the highest concentrations of metal tested suggests to us that zinc and copper play a minor role in *Dictyostelium* resistance to *Legionella* infection.

### Intracellular distribution of free zinc ions in *Dictyostelium* cells

**Figure 10:**
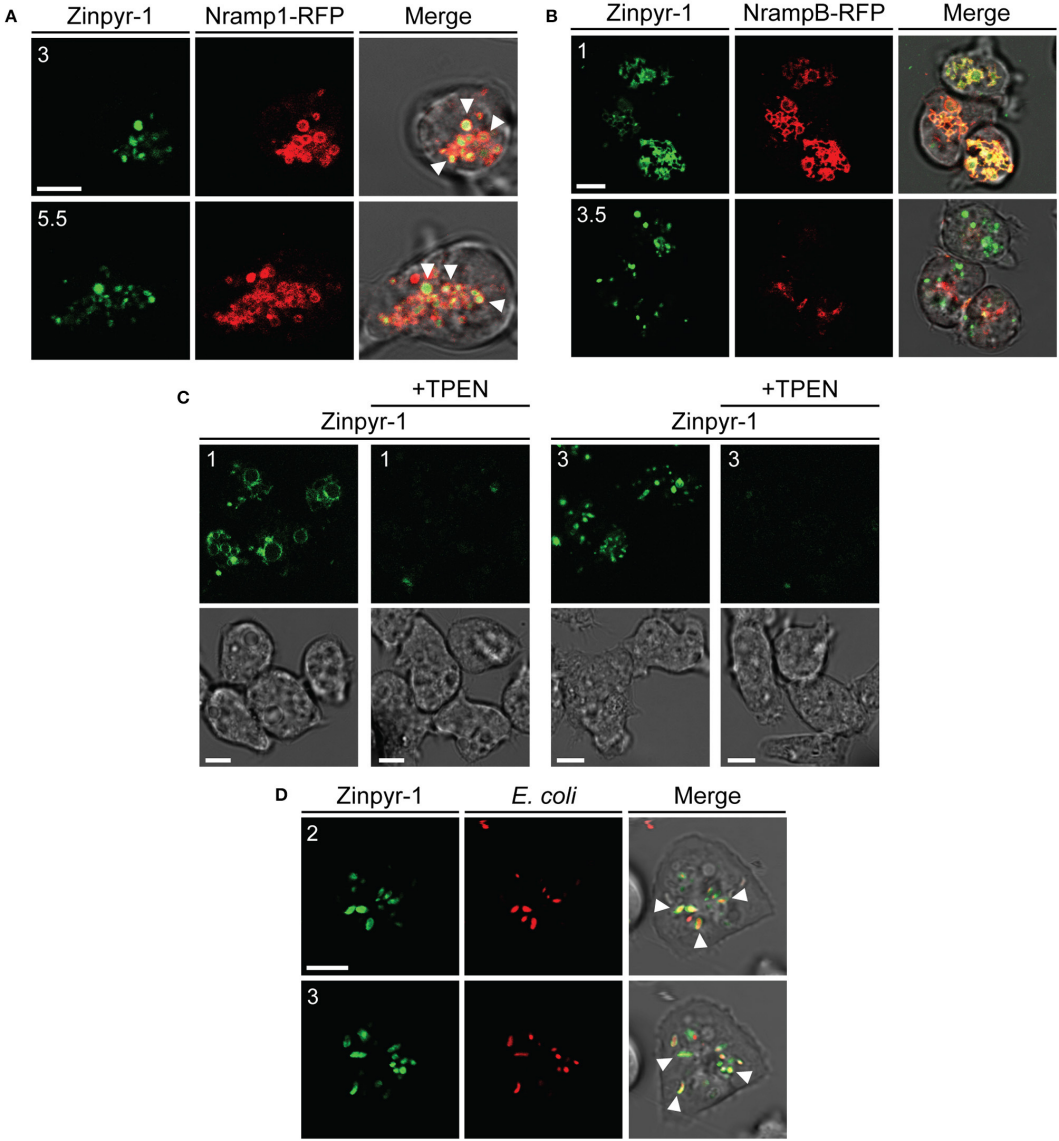
Free zinc ions accumulate in endo-lysosomal vesicles and in the contractile vacuole network. **(A)** AX2 cells producing Nramp1-RFP were washed, resuspended in Soerensen phosphate buffer and incubated with the fluorescent zinc chelator Zinpyr-1 for 30 min. Cells were then washed and resuspended in the same buffer. After plating on glass coverslips, confocal series images were taken. Coincidence of Nramp1-RFP and Zinpyr-1 was detected in almost all vesicles, as indicated by some arrowheads. **(B)** NrampB-RFP producing cells were washed, resuspended in Soerensen phosphate buffer and loaded continuously with Zinpyr-1. Images of cells 30 min after the start of the incubation are shown. The fluorescent zinc reporter labeled the membrane of the contractile vacuole network, identified by NrampB-RFP label, and, faintly, the CV lumen. For all confocal images, red, green fluorescence and merge with corresponding phase-contrast are shown sequentially. **(C)** AX2 cells were washed, resuspended in Soerensen phosphate buffer and incubated with Zinpyr-1 in the presence or not of the zinc intracellular chelator TPEN (25 μM). Green fluorescence (Top) and phase-contrast (Bottom) are shown. In the presence of the chelator, Zinpyr-1 labeling in both contractile vacuole and endo-lysosomal vesicles disappears, indicating that the observed fluorescent signal is not a result of some unspecific effects of Zinpyr-1, but of its interaction with free zinc. **(D)** Living AX2 cells were pulsed with TRITC-labeled *E. coli* and Zinpyr-1 for 30 min, washed and plated on glass coverslips. Confocal section images were taken. Zinpyr-1 green fluorescence co-localized with engulfed bacteria, as indicated by some arrows. The numbers indicate distance from the bottom surface of the cell (in μm). Scale bars: 5 μm.

To better understand if free zinc ions could play a role in predation and infection, we investigated their intracellular localization using the cell-permeable fluorogenic Zn^2+^ reporter Zinpyr-1, which fluoresces in the pH range 3.5 to 8 upon binding Zn^2+^ and is highly selective for the labile Zn^2+^ pool (Burdette et al., 2001; Figueroa et al., 2014). When the probe was administered to AX2 cells producing the iron transporter Nramp1-RFP, the fluorescent signal accumulated in vesicles coated with Nramp1-RFP (Figure 10A). Nramp1 traffics between trans-Golgi and endosomes, including macropinosomes, phagosomes, and endolysosomes, but is not found in postlysosomal vesicles (Peracino et al., 2006). In cells producing the iron transporter NrampB-RFP, we observed also transient labeling of the contractile vacuole (CV) membrane, with a faint labeling inside the vacuole, most evident under continuous Zinpyr-1 loading (Figure 10B). NrampB is localized exclusively in the membrane of the CV tubule network (Peracino et al., 2013). Since Zinpyr-1 under continuous loading failed to label other membranes, including the plasma membrane, the fluorescence in the CV membrane cannot be due to some unspecific effect of Zinpyr-1 diffusing across the membrane bilayer. As additional control, we incubated cells treated with the intracellular membrane-permeable chelator TPEN with Zinpyr-1 under continuous loading. As shown in Figure 10C, the Zinpyr-1 fluorescence is undetectable both at the level of the contractile vacuole and endosomal vesicles. We conclude that the Zinpyr-1 fluorescence in the CV is due to chelation of free zinc ions being transported across the CV membrane. No differences in Zinpyr-1 cellular localization were observed in cells grown in M2 medium ± Zn. In cells incubated with TRITC-labeled *E. coli*, we observed co-localization of the fluorescent probe also with the bacteria in phagosomes (Figure 10D).

Thus, in *Dictyostelium* cells, free zinc ions accumulate in vesicles of the endo-lysosomal pathway, including phagosomes. The transient labeling of the CV membrane and the faint labeling inside the CV suggests that excess free zinc is transported across the CV membrane, where it could be stored or secreted extracellularly. Similar experiments with copper could not be done, as fluorescent reporters for copper are not commercially available.

## Discussion

The high number of copper and zinc transporters in *Dictyostelium* makes difficult a targeted genetic approach to decipher the role of these metals in host-pathogen interactions, in contrast to what has been possible with the Nramp iron transporters (Peracino et al., 2006, 2013). In this paper we have followed an indirect approach, by growing cells under conditions of metal deficiency or overload and then testing the cells for resistance to infection by *L. pneumophila*, which is a very well-studied pathogen for these cells under laboratory conditions (Hägele et al., 2000; Solomon and Isberg, 2000; Peracino et al., 2006; Hilbi et al., 2007; Steinert, 2011; Bozzaro et al., 2013b; Hoffmann et al., 2014).

We used a minimal culture medium (FM Medium) made of amino acids, glucose, a few selected vitamins and trace elements (Franke and Kessin, 1977), whose concentration could be modified. As contamination of pico- to nano-molar traces of metals could derive from reagents (in particular amino acids), glass or other materials (Kay, 2004), the culture medium could not be totally depleted of iron, copper, or zinc, as confirmed by the ICP-MS analysis. The nominally zinc- or copper-free medium still contained, respectively, 7.26 and 4.6% of metal, compared to the standard FM medium. What is more important, the metal concentration in cells growing for more than 20 generations in these nominally zinc- or copper-free media was only slightly reduced compared to cells growing in FM medium, namely of about 15 and 50% for zinc and copper, respectively. Cell growth in media containing 276- or 4,285-fold zinc or copper, respectively, compared to the concentration in the nominally metal-free media, resulted in an increase of only 3- and about 5-fold cellular zinc or copper, respectively, after 24 cell doublings. These data indicate that *Dictyostelium* cells possess very tight regulatory mechanisms of uptake, storing and release of these metals, which can explain why the cell growth rate was absolutely unaffected in media within such a broad range of external zinc or copper concentrations. Only at higher concentrations of copper and zinc, inhibitory and toxic effects on cell growth were detectable, confirming previous results (Burlando et al., 2002; Balbo and Bozzaro, 2008). The high tolerance, at least for copper, could be linked to the activity of one or more MNK Cu-ATPases, whose expression in the plasma membrane is enhanced by addition of salts (Burlando et al., 2002; Hao et al., 2016). For zinc, nothing is known on the cellular localization and activity of the transporters (Sunaga et al., 2008).

In the case of iron, the cellular concentration was reduced to a quarter after 24 h incubation, and about two duplications, in nominally iron-free medium compared to FM medium. We have confirmed that iron is essential for cell growth, with the growth rate being slowed down and cell duplication inhibited within few generations in both nominally iron-free medium and iron overload (Peracino et al., 2013). Iron overload led to formation of very large aggregating streams, which underwent fragmentation. A similar phenotype has been already described for the Nramp1/NrampB double knockout mutant (Peracino et al., 2010), and recalls the streamer mutants, some of which are defective in cGMP phosphodiesterase (Ross and Newell, 1979; Newell and Liu, 1992), suggesting a possible regulation of chemotaxis by iron homeostasis that needs further investigation.

Interestingly, the intracellular growth of *Legionella* was strongly inhibited in cells grown in nominally iron-free medium, but enhanced in cells exposed during growth to 100 or 200 μM iron. Addition of iron to the medium during infection further stimulated *Legionella* growth in iron overloaded cells. The uptake of *Legionella* was also affected by iron, in that the number of cells ingesting *Legionella* was higher in the population grown under iron overload, though the mean number of *Legionella* per cell was constant. Whether this is due to iron being secreted by the iron-overloaded cells, thus stimulating bacterial attachment to the cell surface, is unclear. Increased *Legionella* uptake due to extracellular iron has been recently described in macrophages (O'Connor et al., 2016).

Similar results were obtained with the Nramp1 knockout mutant. The iron transporter Nramp1 mediates iron efflux from the phagosome, and its inactivation increases susceptibility to *L. pneumophila, M. avium*, and *F. tularensis* (Peracino et al., 2006; Buracco et al., 2015; Brenz et al., 2017). Growing the mutant cells in nominally iron-free medium for 24 h strongly inhibited *Legionella* intracellular growth, whereas cell growth under iron overload enhanced its susceptibility to infection to a higher level than in the parental cells, consistent with a higher accumulation of iron in the LCV, due to inactivation of Nramp1 (Buracco et al., 2015). Remarkably, *Legionella* intracellular proliferation in iron overloaded cells was so rapid that already at 48 hpi almost all cells underwent lysis with dispersal of the bacteria in the extracellular milieu. Taken together, these results indicate that a condition of iron starvation is protective for *Dictyostelium* cells against *Legionella* infection whereas high iron cellular content predisposes to rapid infection.

Iron is essential for *Legionella* growth and expression of virulence genes (Robey and Cianciotto, 2002; Cianciotto, 2015; Portier et al., 2015). In *Dictyostelium*, the source of iron for *Legionella* is the macropinosome, where the bacterium is taken up (Peracino et al., 2010). By pumping iron outside the macropinosome via Nramp1, *Dictyostelium* attempts to starve *Legionella* for iron, but *Legionella* can manipulate Nramp1 activity, by avoiding recruitment to the macropinosome of the V-H^+^ ATPase (Peracino et al., 2010), and inhibiting its activity via the Dot/Icm substrate SidK (Xu et al., 2010). The V-H^+^ ATPase is essential for Nramp1-mediated iron efflux (Buracco et al., 2015), thus iron accumulation transforms the macropinosome in a replication-permissive LCV (Steiner et al., 2017). If so, then a condition of iron starvation during cell growth, resulting in less iron being available in the macropinosome, independently of Nramp1 activity, will result in increased resistance of the host cell to *Legionella* infection, while iron overload will increase susceptibility to infection. Recently, an inhibitory effect of iron depletion on *Legionella* infection has been shown in macrophages treated with the iron chelator deferoxamine mesylate during infection or in *Acanthamoeba castellanii* infected in iron-free buffer (O'Connor et al., 2016). In the case of *A. castellanii*, the experimental conditions were different from those used in our experiments, as *A. castellanii* was grown in iron-containing medium and only the infection assay was done in iron-free buffer, which could explain the rather mild effect reported by the authors.

We have shown that *Legionella* proliferation occurs inside the LCV up to cell lysis, with the LCV in close contact to, if not fused with the endoplasmic reticulum (ER) membrane even up to later stages of infection. Association with the ER has been already described at early stages of infection, presumably induced by *Legionella* for acquiring new membrane for the LCV (Fajardo et al., 2004; Lu and Clarke, 2005; Urwyler et al., 2009; Weber et al., 2014; Steiner et al., 2017). Our data suggest that this process sets forth up to later stages.

Interestingly, the LCV membrane becomes gradually permeable between 24 and 48 hpi, allowing diffusion in the vacuole of cytosolic proteins first of lower (27 kDa) and later of higher molecular mass (about 76 kDa), but the bacteria remain packed together and do not egress into the cytosol, suggesting that, though permeable to proteins, the LCV membrane does not allow exit of the bacteria before cell lysis. Vacuole disruption shortly before cell lysis has been reported (Creasey and Isberg, 2012), but the present data suggest that *Legionella* induces permeability of the vacuole in the proliferation phase, possibly to get easy access to nutrients of the host. The dynamics of permeability by using fluorescent probes of increasing molecular size will be reported elsewhere. We failed to detect in the extracellular space clumps of bacteria, which could suggest non-lytic release in LCV's, as described in *A. castellanii* (Berk et al., 1998; Chen et al., 2004; Lau and Ashbolt, 2009).

In sharp contrast to iron, variation in cellular zinc or copper content over a range of, respectively, 3- to 5-fold did not significantly affect timing or extent of *Legionella* intracellular growth. For both, the infection rate was slower, though statistically not significant or borderline, in cells grown in nominally zinc or copper-free medium compared to cells with 5-fold more intracellular copper. If any, this would suggest that a higher intracellular zinc or copper concentration is not toxic for *Legionella*. In the case of zinc, *Dictyostelium* cells with the lowest zinc concentration were blocked at mound stage, suggesting that a suboptimal zinc concentration is deleterious for development. Zinc ions have been shown to enhance pre-stalk to stalk differentiation (Kubohara and Okamoto, 1994) and a family of three ZNT zinc transporters has been shown to be expressed at postaggregative stage in pre-stalk cells (Sunaga et al., 2008).

By using the iron fluorescent reporter calcein, we showed previously that iron is rapidly depleted from endo-lysosomal vesicles via Nramp1 (Buracco et al., 2015). By using the membrane-permeable zinc fluorescent reporter Zinpyr-1, we have now shown that free zinc ions accumulate in vesicles of the endo-lysosomal pathway, including phagosomes, and in the contractile vacuole. Free zinc in other cellular compartments or in the cytosol was below detection with this fluorescent reporter. Zinc transport across the membrane of the contractile vacuole is consistent with the proposed CV role as store or sink for metals (Bozzaro et al., 2013a; Peracino et al., 2013). By using bacteria expressing a copper and a reactive oxygen species biosensor, Hao et al. (2016) showed that copper and reactive oxygen species accumulate in *Dictyostelium* phagosomes upon bacterial phagocytosis. Thus, both zinc and copper are found in *Dictyostelium* phagosomes, consistent with a potential intoxicating role against pathogens, as suggested for macrophages (Babu and Failla, 1990; Wagner et al., 2005; White et al., 2009; Ward et al., 2010; Botella et al., 2011; Chaturvedi and Henderson, 2014; Djoko et al., 2015), though the evidence, at least for zinc, is less clear, and zinc sequestration, likely as it occurs for iron, has been also proposed as defense mechanism (Kehl-Fie and Skaar, 2010; Vignesh et al., 2013).

We have no evidence for phagocytosis inducing a burst of zinc in endocytic vesicles, as shown in macrophages with non-pathogenic bacteria (Botella et al., 2011). We used TRITC-labeled *E. coli*, which are not alive, whereas Botella and coworkers used living bacteria. If this would be the reason, then it would not be phagocytosis *per se* that triggers zinc accumulation, but a cellular response to the ingested living bacteria. Alternatively, the different results can be explained with the different zinc reporters used, Zinpyr-1 in this paper and FluoZin-3 in the Botella et al. (2011) paper. It is worth mentioning that Zinpyr-1 is more specific and selective for free zinc than FluoZin-3 (Figueroa et al., 2014). Further experiments are required to distinguish between these possibilities.

Our data suggest that, in contrast to iron, changes in cellular zinc or copper play a minor role in *Dictyostelium* resistance to pathogens, at least with *L. pneumophila*. Whether this holds true for other pathogens, such as mycobacteria, is under investigation.

A shortcoming of our experimental approach is the apparently very high efficiency of *Dictyostelium* cells to cope with conditions of zinc or copper starvation or overloading. To which extent this is typical of *Dictyostelium* cells or is a common feature of other cells, including macrophages, is unknown. The experimental approach described here offers now the opportunity to identify, in a transcriptomic study, the genes responsible for this high homeostatic adaptation to zinc and copper deprivation or intoxication. From the site of the pathogen, the same approach can be used to identify potential mutants in metal dependent virulence genes.

There is an analogy between the conditions used in this paper and those encountered with humans affected by transition metal deficiency or overloading, due to nutritional or environmental problems. In particular, iron or zinc deficiency is a global nutritional problem, with negative health consequences, including anemia, impaired immune function and neurological diseases. Dietary iron supplementation to prevent micronutrient deficiency in areas with a high burden of infectious diseases is a double–edged weapon, as it can exacerbate morbidity and mortality from infectious diseases (Oppenheimer, 2001; Soofi et al., 2013; Pasricha and Drakesmith, 2016). Similarly, in patients undergoing multiple blood transfusion, the risk of infections is higher, and iron therapy in patients at high risk of infection has been questioned (Ozment and Turi, 2009; Weiss and Carver, 2017). The previous (Peracino et al., 2006; Buracco et al., 2015) and present results with the professional phagocyte *Dictyostelium* support this caution, offering a mechanistic explanation for the iron effects on infection that holds true also for macrophages. Concerning copper, but in particular zinc, further studies are required with macrophages isolated from animals exposed to metal deficiency or overloading. Identifying the genes responsible in *Dictyostelium* for the tight regulation of zinc and copper homeostasis and for their transport in phagosomes can shed some light on cellular zinc and copper store and mobilization, and the genetic basis of antimicrobial metal transport, also in macrophages.

## Author contributions

SBu and SBo designed the experiments; SBu conducted the experiments, acquired, and analyzed the data; BP generated the *Dictyostelium* KO mutants and the plasmids for RFP-fused proteins and helped with some experiments; CA was responsible for the bioinformatic analysis of the metalloproteome. EB cloned the cshA gene, generated, and characterized cells expressing CshA-RFP. SBu and SBo wrote the manuscript; all approved the paper.

### Conflict of interest statement

The authors declare that the research was conducted in the absence of any commercial or financial relationships that could be construed as a potential conflict of interest.

## Supplementary material

Movie S1 3-D reconstruction of AX2 cells infected with GFP-producing *L. pneumophila* 48 hpi. 3-D projections of a single AX2 cell infected with GFP-tagged *L. pneumophila* were generated and animated with the confocal microscope LSM800 software (Zen Software, Carl Zeiss, Inc., Oberkochen, Germany) in.avi format. The movie shows bacteria contained in high number inside the infected cell. Legionellae are not spread in the cell, but are packed together in a “ring”-like structure, presumably around the nucleus.

Figure S1 Plaque formation by AX2 cells previously grown in media depleted or overloaded with iron, zinc, or copper. Ax2 cells were grown in M1 ± Fe for 24 h or in M2 ± Zn or M3 ± Cu for 3 weeks. Cells were then washed, resuspended in Soerensen buffer, and serial dilutions were plated on a lawn of *E. coli* B2, *S. typhimurium*, or *K. aerogenes*, as described in Materials and Methods. Pictures of the appearance and widening of growth plaques were acquired daily with a scanner. Images of the lowest dilution used (10 cells per well) are shown that were acquired 3 or 4 days after plating. No significant differences in growth were observed. Numbers indicate metal concentration in μM (Top) or time in days (on the right).
