# Supplementary figures and images for: Differential Effects of Iron, Zinc, and Copper on Dictyostelium discoideum Cell Growth and Resistance to Legionella pneumophila
Source: Front Cell Infect Microbiol. 2018 Jan 11;7:536. doi: 10.3389/fcimb.2017.00536 (PMC5770829; doi:10.3389/fcimb.2017.00536)

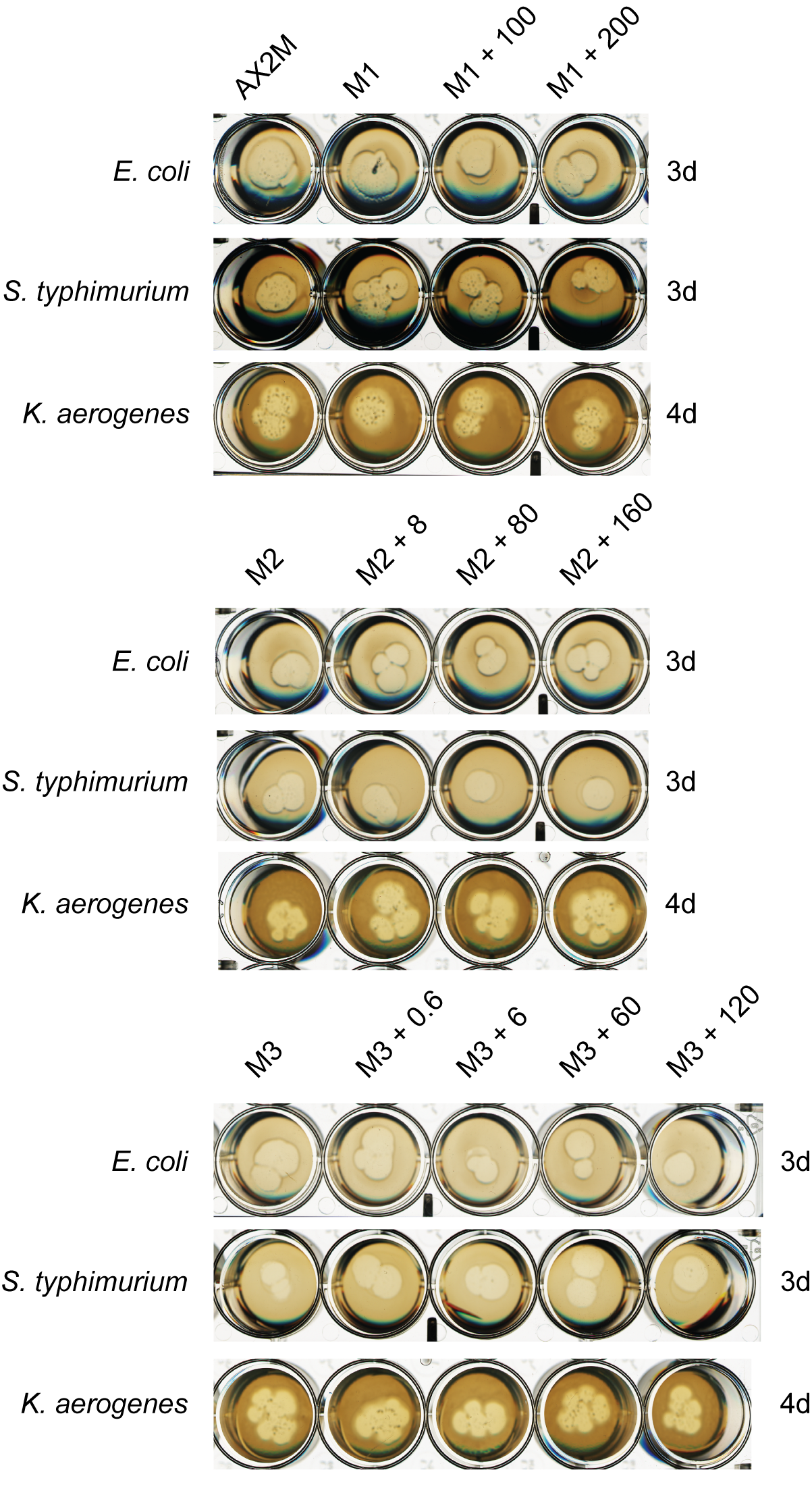

Supplement: Figure S1 — Plaque formation by AX2 cells previously grown in media depleted or overloaded with iron, zinc, or copper. Ax2 cells were grown in M1 ± Fe for 24 h or in M2 ± Zn or M3 ± Cu for 3 weeks. Cells were then washed, resuspended in Soerensen buffer, and serial dilutions were plated on a lawn of E. coli B2, S. typhimurium, or K. aerogenes, as described in Materials and Methods. Pictures of the appearance and widening of growth plaques were acquired daily with a scanner. Images of the lowest dilution used (10 cells per well) are shown that were acquired 3 or 4 days after plating. No significant differences in growth were observed. Numbers indicate metal concentration in μM (Top) or time in days (on the right). [file Image1.TIF]
